# Supplementary material for: Transmission roles of symptomatic and asymptomatic COVID-19 cases: a modelling study
Source: Epidemiol Infect. 2022 Sep 27;150:e171. doi: 10.1017/S0950268822001467 (PMC9588416; doi:10.1017/S0950268822001467)
Supplement: Supplementary file 1 [file S0950268822001467sup001.zip › S0950268822001467sup001.docx]

**Supplementary material: Transmission roles of symptomatic and asymptomatic COVID-19 cases: a modeling study**

**Contents**

[Model structure 2](#_Toc113655543)

[Asymptomatic infections 4](#_Toc113655544)

[Contact function 4](#_Toc113655545)

[Susceptibility-contact-transmissibility transmission process 6](#_Toc113655546)

[B-spline approximations for modeling the transmissibility of symptomatic infections 7](#_Toc113655547)

[The dynamic system of the compartmental model 8](#_Toc113655548)

[Bayesian modeling 9](#_Toc113655549)

[Model assessment 16](#_Toc113655550)

[Supporting results 17](#_Toc113655551)

[Age-varying transmissibility 18](#_Toc113655552)

[Unconfirmed numbers 19](#_Toc113655553)

[Number of newly infected caused by different transmissions 19](#_Toc113655554)

[Simulation studies 20](#_Toc113655555)

[Sensitivity analyses 23](#_Toc113655556)

[Age-varying transmissibility 23](#_Toc113655557)

[Unconfirmed numbers 27](#_Toc113655558)

[Number of newly infected caused by different transmissions 28](#_Toc113655559)

[References 29](#_Toc113655560)

# Model structure

We divided the total population of Zhejiang province into seven age groups: 0-10, 10-20, 20-30, 30-40, 40-50, 50-60, 60+. To consider transmission related to symptomatic and asymptomatic infections among different age groups, our model contained 8 compartments for the $i^{th}$ age group: susceptible population ($S^{i}$), exposed contacts ($E^{i}$), pre-symptomatic cases ($I_{ps}^{i}$, infected but have not yet developed symptoms), symptomatic cases ($I_{s}^{i}$), asymptomatic cases ($I_{a}^{i}$, infected but asymptomatic till confirmed/recovery), and removed/recovery groups ($R_{cs}^{i}$, $R_{ca}^{i}$, $R_{h}^{i}$). We assumed new infections were driven by transmission from compartments of $I_{ps}^{i}$, $I_{s}^{i}$ and $I_{a}^{i}$ in all age groups. Asymptomatic cases ($I_{a}^{i}$) were infections without typical symptoms. These infections were difficult to identify through symptom-based screening; therefore, underreporting in this category of infections was notable. While some asymptomatic cases may have been quarantined through contact tracing, others may have remained unnoticed throughout the entirety of their transmissibility. As such, it is essential to consider unconfirmed asymptomatic cases, also referred as the self-recovered asymptomatic cases ($R_{h}^{i}$ in Figure 1) in modeling. For all age groups, the details of the compartments are given in Table S1, and the state transitions in the compartmental model are given in Figure 1.

Table S1. The definitions of compartments

| Compartments | Meaning |
| --- | --- |
| $S^{i}$ | Represents the individuals who are susceptible to the infectious disease. |
| $E^{i}$ | Represents the individuals who are exposed to the infectious disease but are not infectious. The individuals of $E$ would transfer to $I_{ps}$ or $I_{a}$ with proportion $\alpha_{s}^{i}$ and $\alpha_{a}^{i}$ for different ages after latent period $\mathcal{L}$. |
| $I_{ps}^{i}$ | Represents the symptomatic infectious individuals before symptom onset, which would transfer to $I_{s}^{i}$ after the period of pre-symptomatic transmission $\mathcal{S}_{1}$. |
| $I_{s}^{i}$ | Represents the symptomatic infectious individuals after symptom onset, which would transfer to $R_{cs}^{i}$ after the period of symptomatic transmission $\mathcal{S}_{2}^{i}$. |
| $I_{a}^{i}$ | Represents the asymptomatic infectious individuals, which would transfer to $R_{ca}^{i}$ (confirmed by testing after $\mathcal{A}_{1}^{i}$ days for different ages) or $R_{h}^{i}$ (self-recovered after $\mathcal{A}_{2}$ days) eventually. |
| $R_{cs}^{i}$, $R_{ca}^{i}$ and $R_{h}^{i}$ | Represents the individuals assumed to be noninfectious and immune to the disease in the first wave of Zhejiang epidemic (roughly two months). |

# Asymptomatic infections

Our compartmental model considered the transmission from asymptomatic infections, which were often untraceable in the clinical survey and, therefore, their contribution to population-level transmission would be underestimated. To account for this, we assumed only a proportion of asymptomatic infections were detected ($R_{ca}^{i}$), while others ($R_{h}^{i}$) would be unconfirmed (and not diagnosed) if self-recovery occurred prior to diagnosis. We were able to observe disease confirmation date but not the date of infection for the period from cases becoming infectious to the diagnosis of COVID-19 ($\mathcal{A}_{1}^{i}$) in those with confirmed asymptomatic infection. Based on the virus shedding pattern of asymptomatic infections reported in previous studies,[1–4] we assumed that this period should be less than 30 days, after which virus shedding generally ceases, and infection is no longer detectable through pathogen-specific testing. As such, a uniform prior with an upper bound of 30 days was adopted for$\mathcal{A}_{1}^{i}$. For the period from being infectious to becoming non-infectious (negative conversion of virus shedding) in the undetected asymptomatic infections ($\mathcal{A}_{2}$), we used a more informative log normal prior with a mean of 17 days and standard error of 1.07 based on estimated parameters from a previous study.[1–4] The difference between $\mathcal{A}_{1}^{i} and \mathcal{A}_{2}$ would reflect the intensity of the contact tracing for different ages.

# Contact function

To identify age-varying transmissibility and susceptibility within the compartmental model,[5] we assumed a time-varying curve for the average contact numbers of $i^{th}$ age group with $j^{th}$ age group ($c_{t}^{ij}$). The surveys provided two different strengths of contact (before and during the epidemic). The first survey conducted in 2017 to 2018 in Shanghai was considered as a background of the strength of contact.^16^ The second survey, with the same design, was conducted during the epidemic (from February 1 to 10, 2020).

Although the changing mechanism of social contact between the two cross-sectional surveys was not observed, we assume that it could be described by a function of time (days). We denote the average contact number of the $i^{th}$ with $j^{th}$ age group at time t as $c_{t}^{ij}$, and assume that $c_{t}^{ij}$is a constant at the baseline period of the epidemic and decreases toward the stable level during the outbreak period, as reported cases and the implementations of the nonpharmaceutical interventions increases. In particular, the monotonic decline function $c_{t}^{ij}$ followed the supplied equation[6]:

$c_{t}^{ij}=c_{0}^{ij}\left( \frac{\text{1-}\eta_{ij}}{1+\exp(\lambda_{m}(t-d-\frac{m}{2}))}+\eta_{ij} \right)$ (S1)

where $c_{0}^{ij}$ denotes the maximum contact number at an early stage of the outbreak, $d$ represents the time that $c_{t}^{ij}$ starts declining, $\eta_{ij}$ is the ratio of $c_{0}^{ij}$ to the minimum of $c_{t}^{ij}$, which is $c_{0}^{ij}\eta_{ij}$. We chose $\lambda_{m}$ as $\frac{2\text{log}(99)}{m}$ to ensure $m$ represents the duration of the decreasing process of $c_{t}^{ij}$. An illustrative graph of the contact function is given in Figure S1:

*
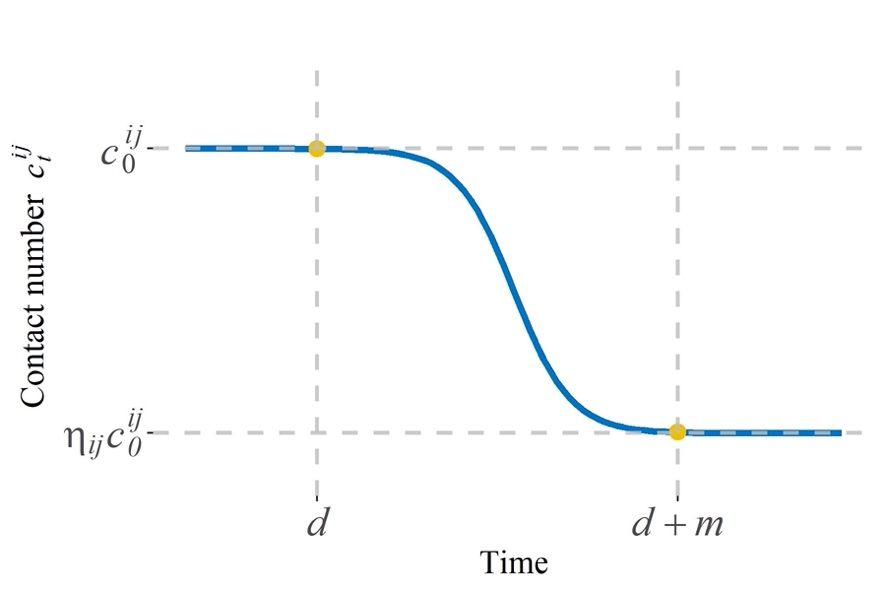
*

*Figure S1. The graph of contact function*

where $c_{0}^{ij}$ and $\eta_{ij}c_{0}^{ij}$ were the contact numbers of $i^{th}$ with $j^{th}$ age group in the baseline period and outbreak period, respectively.

Notice that the contact matrix of Shanghai during the baseline period and outbreak period[7,8] was stratified into 5-year age bands, so the two cross-sectional surveys would not be directly used in modeling of the contact function. We firstly proposed a transformation procedure to stratify them into 10-year age bands. To demonstrate this process, take 0-10 years old as an example. Let $c_{b}^{11}$, $c_{b}^{12}$ be the average contact numbers of 0-5 years old with 0-5 years old and 5-10 years old in the baseline period, respectively. Similarly, let $c_{b}^{21}$, $c_{b}^{22}$ be the average contact numbers of 5-10 years old with 0-5 years old and 5-10 years old in the baseline period, respectively. We calculated the average contact numbers of 0-10 years old with 0-10 years old on baseline period ($c_{b,raw}^{11}$) by:

$$\begin{matrix} c_{b,raw}^{11}=\frac{N_{1}}{N_{1}+N_{2}}\cdot(c_{b}^{11}+c_{b}^{12})+\frac{N_{2}}{N_{1}+N_{2}}\cdot(c_{b}^{21}+c_{b}^{22}), \end{matrix}$$

where $N_{1}$, $N_{2}$ is the number of total populations in 0-5 and 5-10 years old in Zhejiang province, respectively.

After the transformation procedure, let $c_{b,raw}^{ij}$ and $c_{o,raw}^{ij}$ be the transformed average contact numbers of $i^{th}$ age group with $j^{th}$ age group in baseline period and outbreak period. To correct the potential discrepancy in Zhejiang province for the use of the contact numbers in the baseline period and outbreak period of Shanghai, we assume that:

$$c_{0}^{ij}=\beta_{1}c_{b,raw}^{ij},$$

$$\eta_{ij}c_{0}^{ij}=\beta_{2}c_{o,raw}^{ij},$$

where $\beta_{1}$ and $\beta_{2}$ are correction parameters for the contact number in baseline period and outbreak period for Zhejiang province. These two parameters can be later estimated when we specify the compartmental model.

# Susceptibility-contact-transmissibility transmission process

When modeling transmission dynamics of infectious diseases, a key factor to consider is the probability of infection for a susceptible individual given contact with an infectious individual. Here, we separate this concept into two components: transmissibility $(T)$ and susceptibility $(s)$. We define transmissibility $(T)$ as the infectiousness of one case. Theoretically, it is the secondary attack rate in their fully susceptible contacts ($s=1)$. Similarly, we define susceptibility ($s)$ as the probability of acquiring infection from an infectious case ($T=1)$. Therefore, $s=0$ corresponds to a situation in which the susceptible individuals are immune to the disease (this setting was later used to conduct a simulation study to assess possible age-dependent vaccination strategies if a certain age group were to achieve 100% immunity by vaccinations). We assumed that case transmissibility would depend on age and the presence of symptoms. To better quantify transmissibility and susceptibility, we introduced the estimated susceptibility ($s_{i}$) for $i^{th}$ age group from a previous study as a prior in our model.[5] In this setting, the occurrence of a transmission event depends on both transmissibility of the index case and the susceptibility of the contact. The number of secondary cases ($\rho$) from one infectious case at unit time can be calculated as:

$$\rho=s\cdot\text{contact number }\cdot T$$

Let $T_{i,s}$ be the transmissibility of symptomatic infections in $i^{th}$ age group. To capture the age-dependent pattern, B splines basis functions were used to model the variability in age-varying transmissibility smoothly. Given $T_{i,s}$, we assumed that the age-varying transmissibility of asymptomatic infectious individuals was equivalent to $T_{i,a}:=\gamma_{i}T_{i,s}$, where $\gamma_{i}\leq1$, i.e., the transmissibility of asymptomatic cases is no stronger than that of symptomatic cases across different age groups, based on currently available evidence[1,9,10]. The estimated mean of $s_{i}$ and the B spline functions mentioned above are shown in Table S3 and Figure S2 (in the Supplementary Appendix).

# B-spline approximations for modeling the transmissibility of symptomatic infections

Transmissibility was used to quantify the infectiousness of an infected individual. We assume that the infectiousness is related to the virus shedding ability of a case, which may be associated with age[11]. Therefore, we apply a nonparametric estimation approach using the basis expansion approximation. We use three basis functions, as illustrated in Figure S2, to capture possible shapes of transmissibility for different age groups. Specifically, denoting the transmissibility of the $i^{th}$ ages group as $T_{i,s}$, $\mathrm{it}$ can be approximated as:

$T_{i,s}=\sum_{k=1}^{K} \omega_{k}f_{k}(i)$, $\omega_{k}\geq0$, (S2)

where $f_{k}$ are B-spline basis functions (Figure S2) generated by the quadratic piecewise polynomials (with intercept) valued in $\left[ 0, 8 \right]$ through R package **splines**.


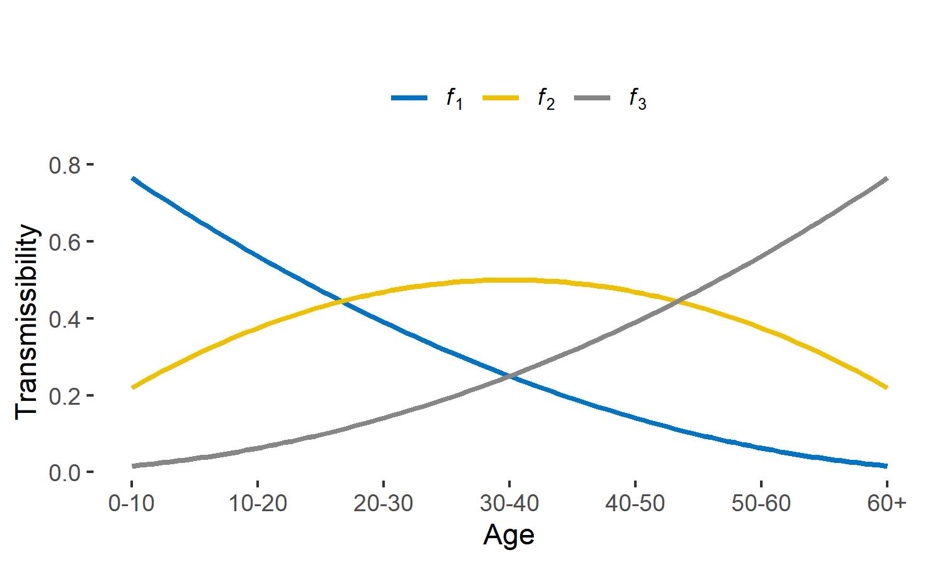


Figure S2. The graph of B-spline.

# The dynamic system of the compartmental model

Let $S^{i}(t), E^{i}(t), I_{ps}^{i}(t), I_{s}^{i}(t), I_{a}^{i}(t), R_{cs}^{i}(t), R_{ca}^{i}(t), R_{h}^{i}(t)$ be the numbers of corresponding compartments at time $t$ in the $i^{th}$ ages group, the dynamic system of the compartments of different ages is assumed to follow:

$$\begin{matrix} \frac{\text{d}S^{i}\left( t \right)}{\text{d}t} & =-S^{i}\left( t \right)\frac{s_{i}\sum_{j} c_{t}^{ij}\left[ T_{j,s}\left( I_{ps}^{j}\left( t \right)+I_{s}^{j}\left( t \right) \right)+T_{j,a}I_{a}^{j}\left( t \right) \right]}{N_{j}}, \\ \frac{\text{d}E^{i}\left( t \right)}{\text{d}t} & =S^{i}\left( t \right)\frac{s_{i}\sum_{j} c_{t}^{ij}\left[ T_{j,s}\left( I_{ps}^{j}\left( t \right)+I_{s}^{j}\left( t \right) \right)+T_{j,a}I_{a}^{j}\left( t \right) \right]}{N_{j}}-\frac{E^{i}\left( t \right)}{\mathcal{L}}, \\ \frac{\text{d}I_{ps}^{i}\left( t \right)}{\text{d}t} & =\frac{E^{i}\left( t \right)}{\mathcal{L}}\alpha_{s}^{i}-\frac{I_{ps}^{i}\left( t \right)}{\mathcal{S}_{1}},\frac{\text{d}I_{s}^{i}\left( t \right)}{\text{d}t}=\frac{I_{ps}^{i}\left( t \right)}{\mathcal{S}_{1}}-\frac{I_{s}^{i}\left( t \right)}{\mathcal{S}_{2}^{i}}, \\ \frac{\text{d}I_{a}^{i}\left( t \right)}{\text{d}t} & =\frac{E^{i}\left( t \right)}{\mathcal{L}}\alpha_{a}^{i}-\frac{I_{a}^{i}\left( t \right)}{\mathcal{A}_{1}^{i}}-\frac{I_{a}^{i}\left( t \right)}{\mathcal{A}_{2}}, \\ \frac{\text{d}R_{cs}^{i}\left( t \right)}{\text{d}t} & =\frac{I_{s}^{i}\left( t \right)}{\mathcal{S}_{2}^{i}}, \\ \frac{\text{d}R_{ca}^{i}\left( t \right)}{\text{d}t} & =\frac{I_{a}^{i}\left( t \right)}{\mathcal{A}_{1}^{i}}, \\ \frac{\text{d}R_{h}^{i}\left( t \right)}{\text{d}t} & =\frac{I_{a}^{i}\left( t \right)}{\mathcal{A}_{2}}, \end{matrix} (S3)$$

where $\alpha_{s}^{i}+\alpha_{a}^{i}=1$. Note that $N_{i}$ and $s_{i}$ are the total population and susceptibility of the $i^{th}$ age group, respectively, and $c_{t}^{ij}$ is the average contact numbers of $i^{th}$ age group with $j^{th}$ age group at time $t$, which is determined by equation (S1) given $c_{b,raw}^{ij}$, $c_{o,raw}^{ij}$, *d, m,* $\beta_{1}$ and $\beta_{2}$. In addition, $T_{i,s}$ is the transmissibility of symptomatic infected individuals in the $i^{th}$ age group specified by equation (S2) given the parameters $\omega_{1}, \omega_{2}, \omega_{3}$. We further use $T_{i,a}=\gamma_{i}T_{i,s}$ as the transmissibility of asymptomatic individuals in $i^{th}$ age group. Moreover, $\mathcal{L,}\mathcal{S}_{1}, \mathcal{A}_{2}$are the average lengths of the latent period, the pre-symptomatic period, and the period from the beginning of asymptomatic transmission to self-recovery for an asymptomatic infection, respectively. Likewise, $\mathcal{S}_{2}^{i}$ represents the mean duration of the period of symptomatic transmission and $\mathcal{A}_{1}^{i}$ represents the period between the beginning of asymptomatic transmission and the diagnosis of asymptomatic infection in the $i^{th}$ age group.

Based on the equation system (S3), we can calculate the infected numbers caused by symptomatic and asymptomatic transmission for different ages, which is achieved by defining new compartments:

$$\frac{dE_{s}^{j}\left( t \right)}{dt}=\sum_{i} S^{i}\left( t \right)\frac{s_{i}T_{j,s}c_{t}^{ij}\left( I_{ps}^{j}\left( t \right)+I_{s}^{j}\left( t \right) \right)}{N_{j}}, \left( S4 \right)$$

$$\frac{dE_{a}^{j}\left( t \right)}{dt}=\sum_{i} S^{i}\left( t \right)\frac{s_{i}T_{j,a}c_{t}^{ij}I_{a}^{j}\left( t \right)}{N_{j}}, (S5)$$

where $E_{s}^{j}\left( t \right)$ and $E_{a}^{j}\left( t \right)$ represent the number of the infected individuals caused by symptomatic and asymptomatic transmission from $j^{th}$ age group, respectively. Similarly, we define $E_{s}\left( t \right)=\sum_{j} E_{s}^{j}\left( t \right)$ and $E_{a}\left( t \right)=\sum_{j} E_{a}^{j}\left( t \right)$ as the number of infected individuals caused by symptomatic and asymptomatic transmissions. We also use the ratios $\frac{E_{a}}{E_{s}+E_{a}}, \frac{E_{s}^{j}}{E_{s}}$ and $\frac{E_{a}^{j}}{E_{a}}$to quantify the burden of asymptomatic transmission, the contribution of $j^{th}$age group among symptomatic and asymptomatic transmissions, respectively.

# Bayesian modeling

A Bayesian paradigm provides a coherent probabilistic approach to introduce reasonable uncertainties into the modeling of epidemic process[12]. In particular, let

$$\begin{matrix} Z(t) & :=(S^{i}(t),E^{i}(t),I_{ps}^{i}(t),I_{s}^{i}(t),I_{a}^{i}(t),R_{cs}^{i}(t),R_{ca}^{i}(t),R_{h}^{i}(t))_{i=1:7}, \\ \Omega: & =(\mathcal{L},\mathcal{S}_{1},\mathcal{A}_{2},d,m,\beta_{1},\beta_{2},\omega_{1},\omega_{2},\omega_{3},(s_{i},\alpha_{a}^{i},\mathcal{S}_{2}^{i},\mathcal{A}_{1}^{i},\gamma_{i})_{i=1:7}), \end{matrix}$$

where $\Omega$ is the collection of all parameters needed to be estimated. As mentioned above, we assume that$\mathcal{C}_{b,raw}:=(c_{b,raw}^{ij})_{i,j=1:7}$, $\mathcal{C}_{o,raw}:=(c_{o,raw}^{ij})_{i,j=1:7}$, the estimated means of the age-varying susceptibility ${(s_{i,\mu})}_{i=1:7}$and $N:=(N_{i})_{i=1:7}$ are known, which are shown in Table S2-S5:

Table S2. Transformed contact matrix (10-year age bands) during the baseline period ($\mathcal{C}_{b,raw})$, consisting of the average number of contacts per day.

| Age | | Age of contact | | | | | | |
| --- | --- | --- | --- | --- | --- | --- | --- | --- |
|  |  | 0-10 | 10-20 | 20-30 | 30-40 | 40-50 | 50-60 | 60+ |
| Age of participant | 0-10 | 8.31 | 0.93 | 0.69 | 2.11 | 0.35 | 0.57 | 1.34 |
|  | 10-20 | 0.48 | 19.17 | 1.35 | 3.13 | 2.51 | 1.14 | 1.06 |
|  | 20-30 | 0.16 | 0.21 | 4.38 | 5.66 | 5.33 | 3.28 | 1.77 |
|  | 30-40 | 0.60 | 0.45 | 3.14 | 6.88 | 5.28 | 3.20 | 1.74 |
|  | 40-50 | 0.12 | 0.68 | 2.80 | 5.44 | 5.34 | 2.69 | 1.99 |
|  | 50-60 | 0.17 | 0.24 | 2.34 | 3.91 | 3.91 | 3.80 | 3.96 |
|  | 60+ | 0.13 | 0.13 | 0.59 | 1.22 | 1.53 | 1.90 | 6.12 |

Table S3. Transformed contact matrix (10-year age bands) during the outbreak period $(\mathcal{C}_{o,raw})$, consisting of the average number of contacts per day.

| Age | | Age of contact | | | | | | |
| --- | --- | --- | --- | --- | --- | --- | --- | --- |
|  |  | 0-10 | 10-20 | 20-30 | 30-40 | 40-50 | 50-60 | 60+ |
| Age of participant | 0-10 | 0.02 | 0.00 | 0.28 | 1.41 | 0.16 | 0.07 | 0.00 |
|  | 10-20 | 0.11 | 0.22 | 0.48 | 0.53 | 1.10 | 0.43 | 0.37 |
|  | 20-30 | 0.07 | 0.07 | 0.24 | 0.16 | 0.37 | 0.65 | 0.21 |
|  | 30-40 | 0.40 | 0.17 | 0.17 | 0.71 | 0.35 | 0.16 | 0.52 |
|  | 40-50 | 0.21 | 0.56 | 0.26 | 0.33 | 0.77 | 0.34 | 0.51 |
|  | 50-60 | 0.06 | 0.06 | 0.42 | 0.17 | 0.20 | 0.52 | 0.42 |
|  | 60+ | 0.05 | 0.06 | 0.08 | 0.24 | 0.08 | 0.07 | 0.83 |

Table S4. The estimated mean of the age-varying susceptibility^22^ ${(s_{i,\mu})}_{i=1:7}$

| Age | 0-10 | 10-20 | 20-30 | 30-40 | 40-50 | 50-60 | 60+ |
| --- | --- | --- | --- | --- | --- | --- | --- |
| $s_{i,\mu}$ | 0.40 | 0.38 | 0.79 | 0.86 | 0.80 | 0.82 | 0.81^[[1]](#footnote-1)^ |

Table S5. The age structure of Zhejiang province[13] $(N)$

| Age | 0-10 | 10-20 | 20-30 | 30-40 | 40-50 | 50-60 | 60+ |
| --- | --- | --- | --- | --- | --- | --- | --- |
| Population | 4811720 | 5969042 | 9417394 | 9632456 | 10201561 | 6836085 | 7558633 |

Let $D_{t}^{i,1}$ be the number of daily new symptomatic infected individuals, as defined by the onset of symptoms, at time $t$ in $i^{th}$ age group, and $D_{t}^{i,2}$ be the number of daily new confirmed asymptomatic infected individuals at time $t$ in $i^{th}$ age group. Similarly, $\mathcal{D}_{t}^{i,g}(Z(0),\Omega) (g=1,2$) represents the corresponding fitted values of the epidemic equation (S3) given through the initial value $Z(0)$ and the parameters $\Omega$, where $Z(0)$ is specified by the initial cases of symptom onset on January 8th, 2020 and population numbers in Table S4. For a flexible adjustment of overdispersion of the new numbers, we use a negative binomial mass as the loss function for $D_{t}^{i,g}$:

$\begin{matrix} \text{Lik}=\Pi_{t=1}^{T}\Pi_{i=1}^{7}\Pi_{g=1,2}\text{Nb}(D_{t}^{i,g}|\mathcal{D}_{t}^{i,g}(Z(0),\Omega),v_{g}), \end{matrix}$

where Lik means the likelihood of data, $T$ is the time length of the observed data and $\text{Nb}(u,v)$ represents the mass function of the negative binomial distribution with mean $u$ and size $v$. We illustrate our data for modeling fitting in Figure S3. The note of these parameters is given in Table S6.

Table S6. The note of parameters

| Parameters | Meaning | Reference value | Prior distribution |
| --- | --- | --- | --- |
| $\mathcal{L}$ | Latent period | 2 days[14,15] | $\log N(2,\sigma)$ |
| $\mathcal{S}_{1}$ | The period of pre-symptomatic transmission | 3-4 days[16,17] | $\log N(3,\sigma)$ |
| $\mathcal{S}_{2}^{i}$ | The period of symptomatic transmission | Null | $IG(a_{i}, b_{i})$ |
| $\mathcal{A}_{1}^{i}$ | The period from the beginning of asymptomatic transmission to diagnosis for an asymptomatic infection | Null | $Unif(\frac{b_{i}}{a_{i}}, 30)$ |
| $\mathcal{A}_{2}$ | The period from the beginning of asymptomatic transmission to recovered for an asymptomatic infection | 17 days (standard deviation 1.07)[4] | $\log N(\text{17},\text{1.07})$ |
| $s_{i}$ | The susceptibility for different age groups | Corresponding value in Table S4 | $N_{[0,1]}(s_{i,\mu},\sigma_{s})$ |
| $\alpha_{a}^{i}$ | The ratio to be the asymptomatic infection after latent period for different age groups | Null | $Beta(2,2)$ |
| $\gamma_{i}$ | The ratio of the transmissibility of asymptomatic infection to symptomatic infection for different age groups | Null | $Beta(2,2)$ |
| *d, m* | The time that the contact starts declining and the duration of the decreasing process | Null | $\mathrm{Unif}(0, 30)$ |
| $\beta_{1},\beta_{2}$ | Correction parameter for the contact matrix | Null | $N_{[0, 2]}(1, 0.2)$ |
| $\omega_{1},\omega_{2},\omega_{3}$ | The coefficients of the B-splines in Figure S2 | Null | $N_{[0,\infty]}(0, 0.1)$ |
| $v_{1}, v_{2}$*.* | The parameters controlling the degree of overdispersion | Null | $Gamma(2, 1)$ |

Here $log N\left( \mu,\sigma\right)$ represents a log-normal distribution with log-mean $\text{log}\left( \mu\right)$ and log-standard deviation $\text{log}\left( \sigma\right)$. $N_{\left[ a, b \right]}\left( \mu,\sigma\right)$ represents a truncated normal distribution valued in $\left[ a,b \right]$ with mean $\mu$ and standard deviation $\sigma$. $\mathrm{IG}\left( \cdot, \cdot\right), Unif(\cdot, \cdot), Beta(\cdot, \cdot)$and $Gamma(\cdot, \cdot)$ denote the inverse Gamma distribution, Uniform distribution, Beta distribution, and Gamma distribution with their corresponding parameters, respectively. $Beta(2, 2)$, $Unif(0, 30), and Gamma(2, 1)$are flat priors for corresponding parameters, respectively. Moreover, we set $\sigma=2$*,* i.e. chose flat prior distributions for $\mathcal{L,}\mathcal{S}_{1}.$ According to the study of susceptibility for different ages^22^, we set $\sigma_{s}=0.075$ as the standard deviation of the truncated normal distributions for all age groups. The weakly informative prior distributions of ${(\gamma_{i})}_{i=1:7}$, $\beta_{1},\beta_{2}$, and $\omega_{1},\omega_{2},\omega_{3}$ are chosen to regularize the estimates of parameters for the stability of model fitting.

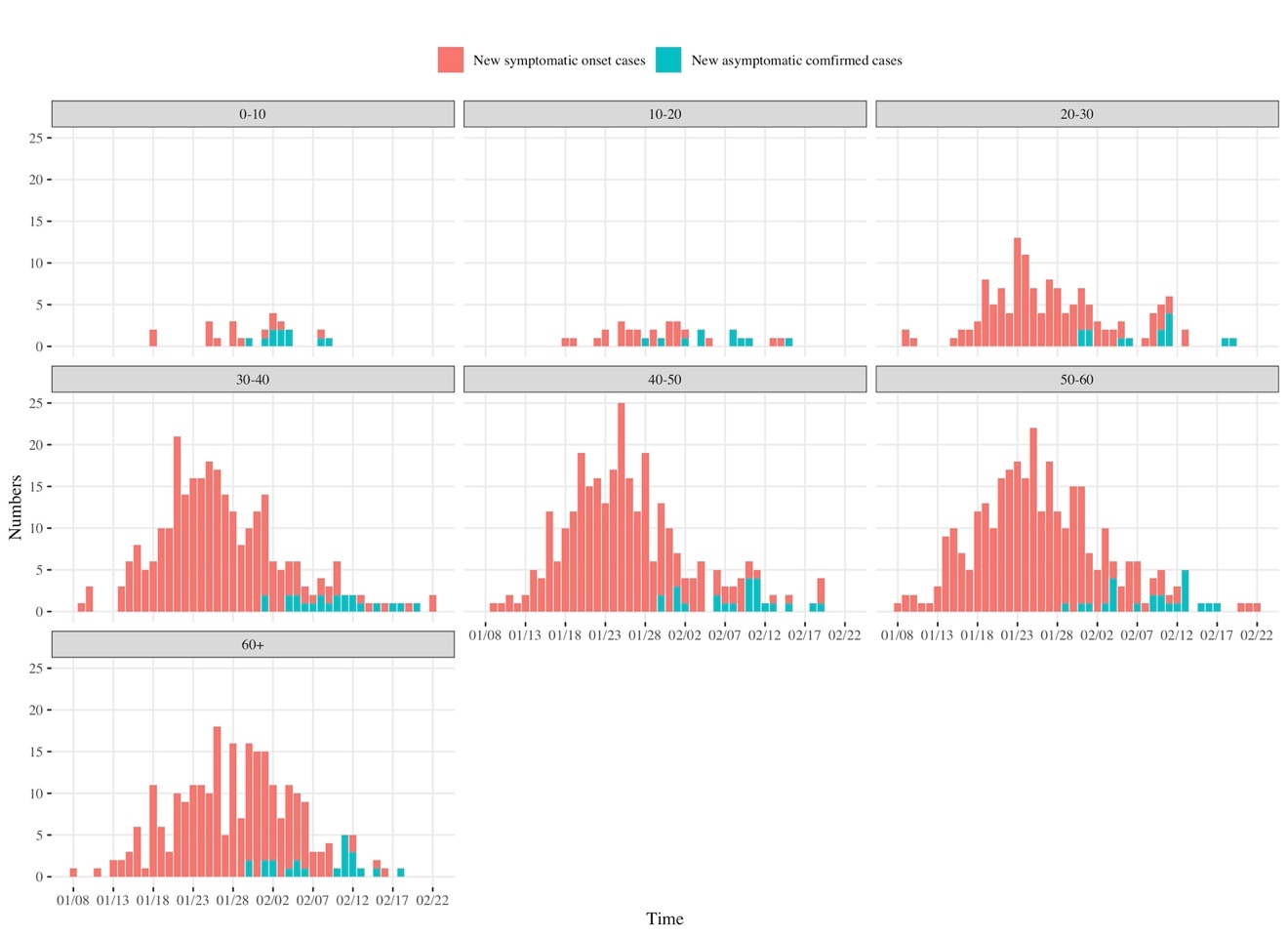


Figure S3. The new numbers of corresponding cases in seven age groups.

Hyper-parameters of the prior distribution of $\mathcal{S}_{2}^{i}$ are estimated from the clinical data of Zhejiang province for different age groups. To be specified, let $W_{l}^{i}$ be the observed period of symptomatic transmission for the $l^{th}$ individual in the $i^{th}$ age group, we assume an exponential mass for $W_{l}^{i}$, where $l$*=1, …,*$L_{i}$, equipped with the Jeffreys' prior for the inverse of rate parameter $\lambda_{i}$ of an exponential distribution[18], then the posterior distribution of $\lambda_{i}$ is given as:

$$\pi(\lambda_{i}|W_{1}^{i},...,W_{L_{j}}^{i})\propto\frac{1}{\lambda_{i}}\prod_{l=1}^{L_{i}} \frac{1}{\lambda_{i}}e^{-\frac{W_{l}^{i}}{\lambda_{i}}}=IG(L_{i}, \sum_{l=1}^{L_{i}} W_{l}^{i}). (S6)$$

We use the posterior distribution of $\lambda_{i}$ as the prior distribution of $\mathcal{S}_{2}^{i}$, i.e. $a_{i}=L_{i}$ and $b_{i}=\sum_{l=1}^{L_{i}} W_{l}^{i}$. The estimated hyper-parameters are also used in the construction of the lower bound of the prior of $\mathcal{A}_{1}^{i}$. We assume that the asymptomatic infections are harder to detect than symptomatic infections after the symptom onset, i.e. $\mathcal{A}_{1}^{i}$ is longer than the mean of $\mathcal{S}_{2}^{i}$, while the upper bound of $\mathcal{A}_{1}^{i}$ is chosen as 30 since most of the asymptomatic infections will self-recover after one month[1–4]. For the period $\mathcal{A}_{2}$, we utilize the period from diagnosis to the first negative conversion for asymptomatic infections reported in a previous clinical study [1–4] ( a median of 17 days with a standard error of 1.07 days) to specify an informative prior distribution.


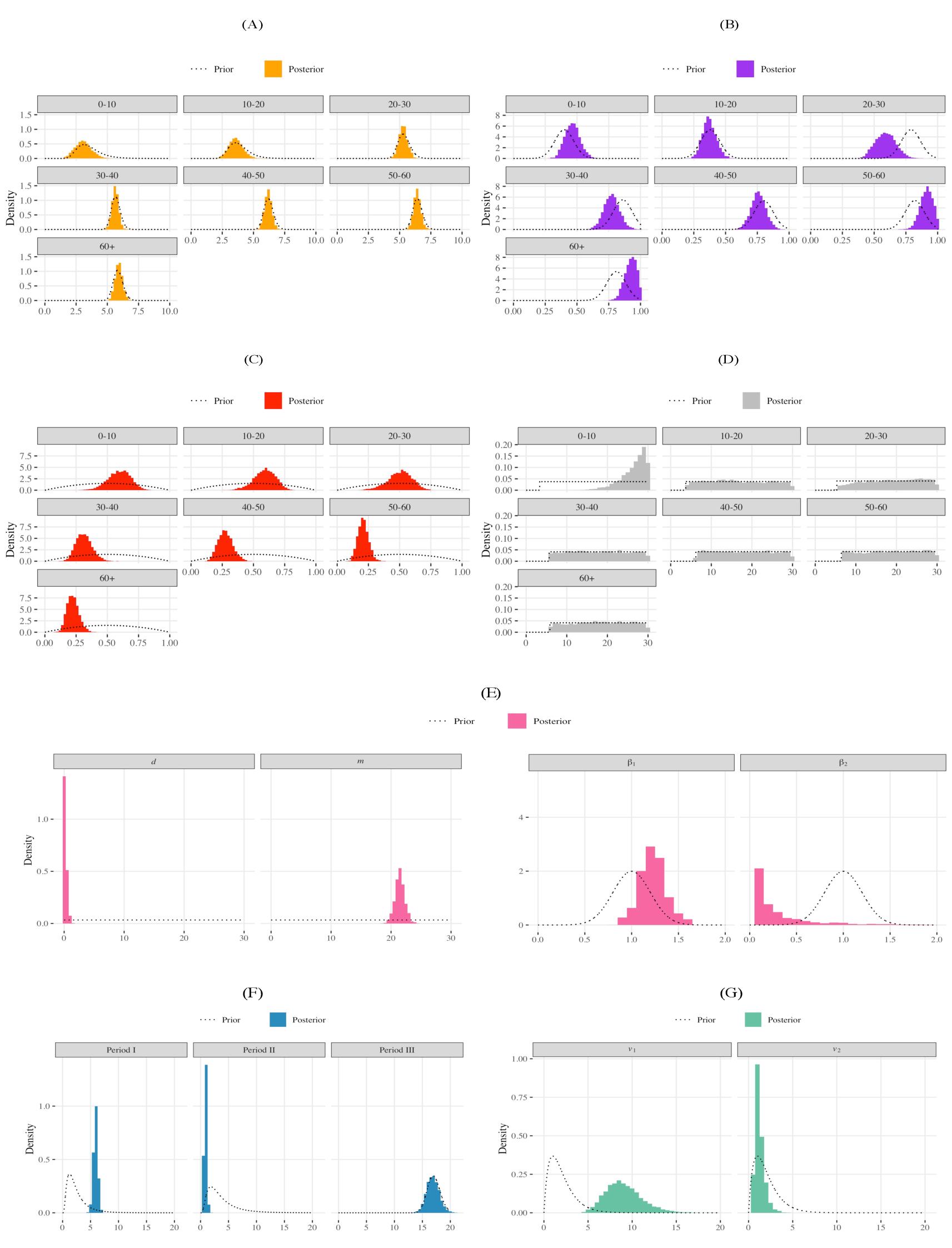

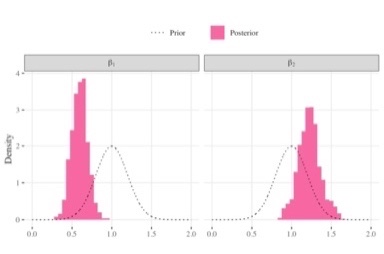


Figure S4. The posterior distributions of the parameters compared with their priors, where A, B, C, D denote $\mathcal{S}_{2}^{i},$ $s_{i}, \alpha_{a}^{i}$ and $\gamma_{i}$, E denotes the parameters relating to the contact function, F denotes $\mathcal{L,}{\mathcal{S}_{1}\mathcal{,A}}_{2}$, and G denotes $v_{1}, v_{2}$, respectively.

Regarding the computing algorithm, we use a random walk Metropolis-within-Gibbs algorithm[19,20] combined with mixture transfer variance[20] to sample the posterior distribution of the parameters of our compartmental model. A parallel computing procedure implemented by R package snowfall[21] is used to efficiently generate the burn-in samples of the posterior distribution. After the convergence of MCMC, a minimum of 4000 post-burn-in samples were collected to construct the posterior distributions and credible intervals. Except for the parameters relating to the transmissibility (which have been shown in the main text), we presented the posterior distributions of the remaining parameters in Figure S4. Subsequently, the dynamic curve of different compartments considering the posterior uncertainty could be achieved by equations (S3), (S4) and (S5) given the initial condition. These reconstructed results would be used for an illustration of the outbreak to provide Insight into the invisible dynamics of the epidemic.

# Model assessment

We treat our structured epidemic data from January 8th to February 15th, 2020 as a training set and use the data from February 16th to February 22nd, 2020 as a testing set to evaluate the prediction behavior of our compartmental model. As an illustration of our model fitting, we present the fitting and prediction of the daily total new numbers of symptom onset for symptomatic infections and confirmed case for asymptomatic infections, and illustrate the predicted 95% intervals of the confirmed cases for different age groups in Figure S5. To measure the goodness-of-fit of our compartmental model, we use the sum of squares of residuals as the discrepancy criterion:

$$\mathrm{Dis}\left( D^{g},\Omega\right):=\sum_{t=1}^{T_{t}} \left( {D_{t}^{g} \mathcal{- D}}_{t}^{g}\left( Z\left( 0 \right),\Omega\right) \right)^{2},$$

where $D^{g}=\left( D_{1}^{g},...,D_{T_{t}}^{g} \right)$, $D_{t}^{g}=\sum_{i=1}^{7} D_{t}^{i,g}, \mathcal{D}_{t}^{g}\left( Z\left( 0 \right),\Omega\right)= \sum_{i=1}^{7} \mathcal{D}_{t}^{i,g}\left( Z\left( 0 \right),\Omega\right)$ and $T_{t}=T-7$. We use the posterior predictor assessment of model[22] via the realized discrepancy $\mathrm{Dis}\left( D^{g},\Omega\right)$, and the Bayesian p-value for the goodness-of-fit test is calculated through the generated MCMC samples.

Our results show a good fit to the daily new numbers of symptom onset cases (Figure S5 (A)) and daily new confirmed asymptomatic infections (Figure S5 (B)). The Bayesian p-values of the goodness-of-fit test are 0.451 and 0.180, respectively, implying that there is no evidence against the fitted curves. Specially, the observed numbers in the prediction period (Figure S5) are all covered by the credible bands, which indicate the reliability of the proposed compartmental model and simulation results.


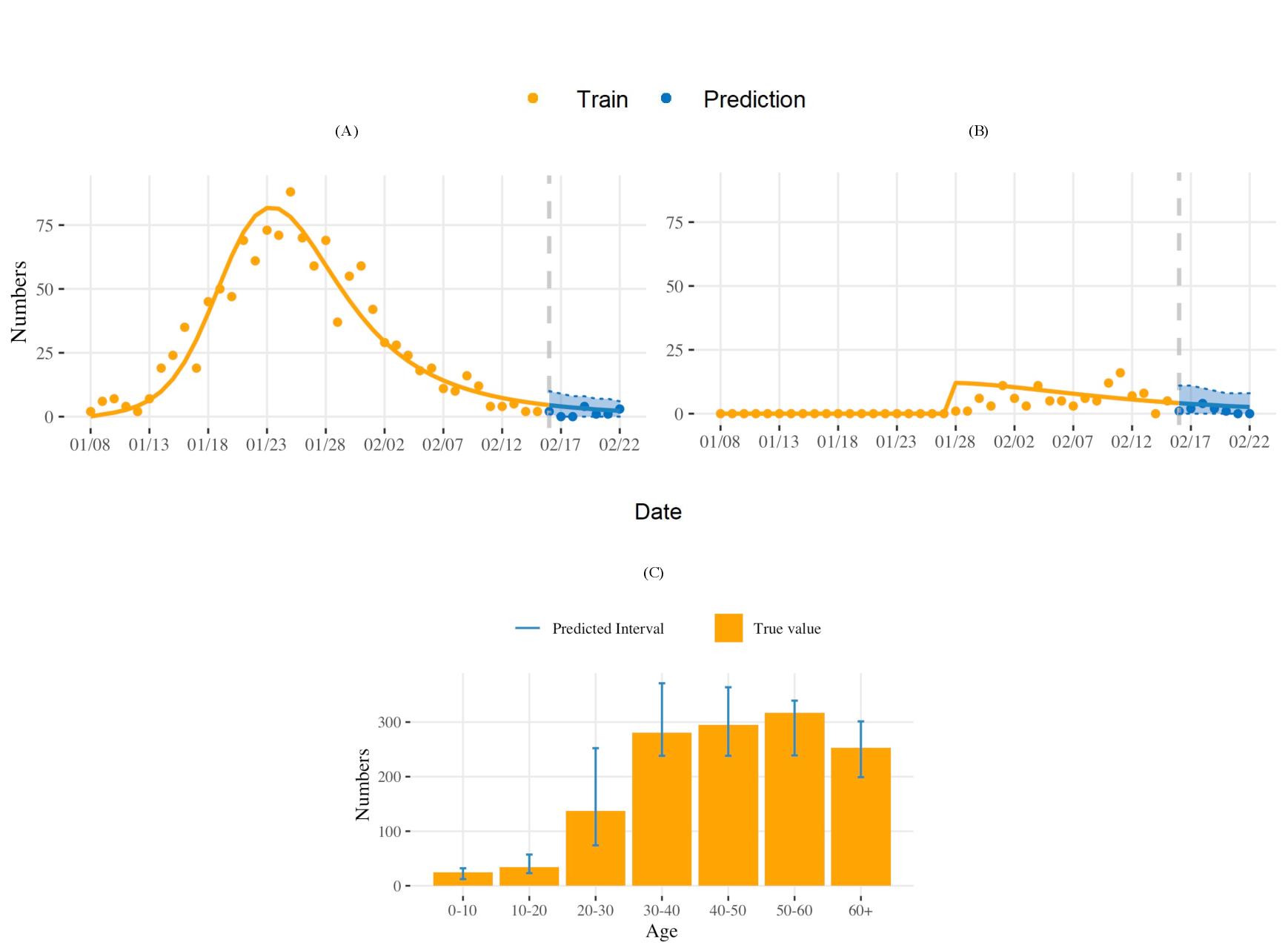


Figure S5. The daily total new numbers of symptom onset for symptomatic infections (A) and confirmed cases for asymptomatic infections (B) for all age groups from January 8th to February 22nd, 2020, and the numbers of observed cases up to February 22nd with 95% predicted intervals of our model (C)**.** The fitting curve from January 8th to February 15th, 2020 and the predicted numbers with a 95% credible band from February 16th to February 22nd, 2020 are presented, where the fitting and predicted numbers are calculated by the means of posterior distributions.

# Supporting results

## Age-varying transmissibility

The estimated age-varying transmissibility of asymptomatic and symptomatic infections of Figure 2 are given in Table S7 and Figure S6.

Table S7. The estimated transmissibility and the ratios of the medians of their posterior distributions ($\frac{T_{i,a}}{T_{i,s}}$) in different age groups

|  | The transmissibility of symptomatic infection ($T_{i,s}$) | The transmissibility of asymptomatic infection ($T_{i,a}$) | Ratio of transmissibility $T_{i,a}/T_{i,s}$(%) |
| --- | --- | --- | --- |
| 0-10 | 0.043 (0.014, 0.123) | 0.016 (0.003, 0.071) | 35.70 |
| 10-20 | 0.058 (0.029, 0.116) | 0.021 (0.006, 0.062) | 35.73 |
| 20-30 | 0.078 (0.050, 0.124) | 0.024 (0.008, 0.065) | 30.35 |
| 30-40 | 0.107 (0.077, 0.149) | 0.026 (0.011, 0.077) | 24.42 |
| 40-50 | 0.144 (0.107, 0.186) | 0.034 (0.015, 0.093) | 23.38 |
| 50-60 | 0.189 (0.142, 0.241) | 0.055 (0.020, 0.152) | 29.04 |
| 60+ | 0.243 (0.182, 0.309) | 0.132 (0.036, 0.224) | 54.33 |


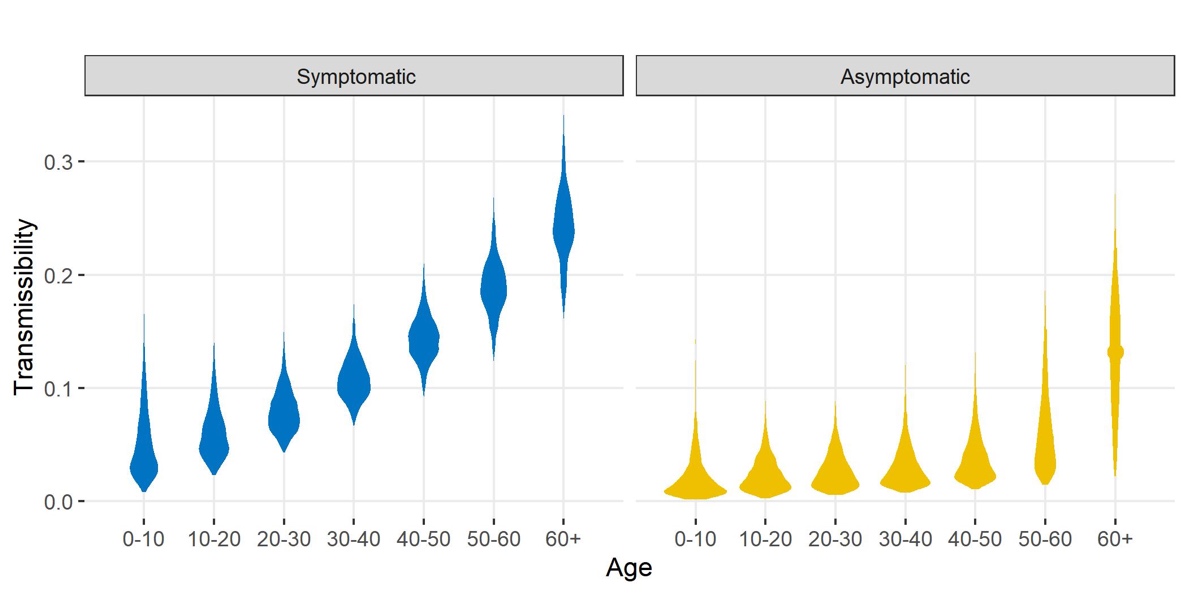


*Figure S6. The eye-plot of the posterior distributions of transmissibility for seven age groups.*

Typically, the estimated transmissibility was identified based on the reported susceptibility, which implied elder persons were more susceptible than the younger people[5]. This relative measure would be helpful for us to the evaluate the true intensity of shedding virus for infectious individuals.

## Unconfirmed numbers

We present the observed numbers of symptomatic cases, asymptomatic cases and the estimated unconfirmed numbers of asymptomatic infections for different age groups in Table S8, which are used for the calculation of Figure 3.

Table S8. The infected individuals until February 22^nd^, 2020 for different age groups

|  | Observed numbers of symptomatic cases | Observed numbers of asymptomatic cases | Estimated unconfirmed number of asymptomatic infections |
| --- | --- | --- | --- |
| 0-10 | 15 | 10 | 12.67 (7.33, 20.44) |
| 10-20 | 24 | 10 | 22.66 (11.67, 38.68) |
| 20-30 | 123 | 14 | 90.39 (39.12, 165.37) |
| 30-40 | 259 | 22 | 75.21 (41.83, 133.10) |
| 40-50 | 272 | 23 | 63.21 (34.72, 108.29) |
| 50-60 | 294 | 23 | 44.19 (25.55, 70.60) |
| 60+ | 231 | 22 | 40.61 (24.05, 68.13) |
| Total | 1218 | 124 | 354.30 (239.11, 519.30) |

## Number of newly infected caused by different transmissions

The estimated results related to Figure 4 and 5 are given in Table S9 and S10, which are estimated from equations (S4) and (S5).

Table S9. The newly infected numbers caused by symptomatic $E_{s}\left( t \right)$ and asymptomatic transmission $E_{a}\left( t \right)$during different time period

|  | Infected numbers caused by symptomatic transmission | Infected numbers caused by asymptomatic transmission | Proportion of the asymptomatic transmission (%) |
| --- | --- | --- | --- |
| 01/08-01/12 | 74.18 (59.53, 92.56) | 6.25 (3.24, 10.85) | 7.77 |
| 01/13-01/17 | 415.47 (321.21, 527.94) | 57.85 (29.21, 100.43) | 12.22 |
| 01/18-01/22 | 739.91 (656.73, 844.47) | 110.71 (61.06, 179.17) | 13.02 |
| 01/23-01/27 | 276.81 (195.25, 370.57) | 46.11 (25.41, 75.09) | 14.28 |
| 01/28-02/01 | 38.01 (20.84, 64.27) | 7.26 (3.57, 13.05) | 16.03 |

Table S10. The infected numbers caused by different age groups until February 1^st^, 2020

|  | Contribution rate for symptomatic transmission (%) | Contribution rate for asymptomatic transmission (%) |
| --- | --- | --- |
| 0-10 | 0.09 (0.03, 0.32) | 0.49 (0.09, 2.59) |
| 10-20 | 0.41 (0.19, 0.90) | 1.99 (0.42, 7.84) |
| 20-30 | 3.77 (2.55, 5.70) | 9.44 (2.62, 27.74) |
| 30-40 | 18.53 (15.19, 22.75) | 16.52 (5.84, 40.98) |
| 40-50 | 21.65 (18.70, 24.92) | 14.74 (5.55, 37.32) |
| 50-60 | 28.63 (25.19, 32.16) | 16.49 (5.67, 40.69) |
| 60+ | 26.55 (21.65, 31.36) | 31.73 (10.19, 56.10) |

# Simulation studies

Simulation studies are conducted to further investigate the dynamic changes in transmission burden over time during a prolonged epidemic (Figure S7). When the duration of the decreasing process of contact function (represented by “$m$” in Figure S1) was prolonged by two weeks and each individual’s daily contact number increased by one person during the outbreak period, we found a slower decreasing trend in daily new cases. Furthermore, the proportion of asymptomatic infections contributing to the spread of the virus increased during the early outbreak and period in which daily new cases are decreasing (Figure S7, scenario 1), which implies that although the early outbreak was mostly contributed to by symptomatic infections, asymptomatic transmission would increase rapidly thereafter and continue to rise along with the constant outbreak of COVID-19.

We further proposed two more simulations (Figure S7, scenario 2 and scenario 3). In order to promote the generalizability of our model to better guide interventions in other regions, we let the time needed for tracing the symptomatic cases shrink 50% based on scenario 1 above (scenario 2), and then let one jurisdiction have limited ability to trace contacts, thereby being generally unable to find asymptomatic cases based on scenario 2 (scenario 3). In scenario 2, quicker isolation effectively suppressed the spread of COVID-19, while the burden of asymptomatic transmission increased. In scenario 3, asymptomatic transmission dominated the total transmission when the number of daily new infected cases was still considerable.

Overall, we found either symptomatic or asymptomatic transmission could dominate the outbreak (Figure S7). If strong controlling measures are solely implemented on symptomatic cases, the unrestricted asymptomatic transmission will likely prolong the needed time of interventions and potentially delay reopening.


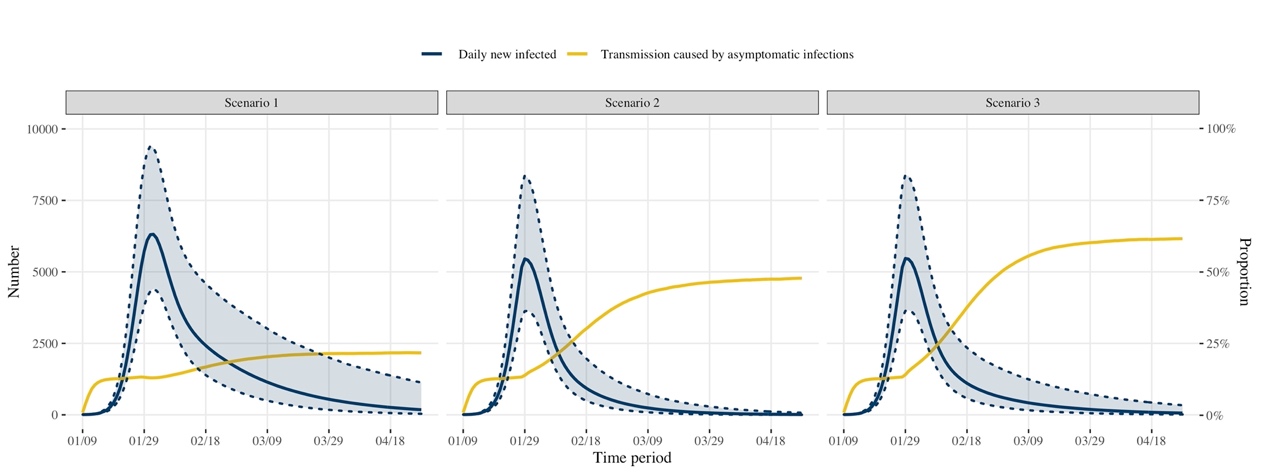


*Figure S7. The simulated daily new infected numbers and their proportion of new infected cases caused by asymptomatic transmission (from January 8th to April 27^th^, 2020), where* ***scenario 1*** *assumed the estimated duration of the decreasing process of contact matrix was prolonged by two weeks and average contact numbers per day for each age group was increased by 1 during the lockdown period.* ***Scenario 2*** *assumed the duration between symptom onset and confirmation by nucleic testing (represented by* $\mathcal{S}_{2}^{i}$*) for symptomatic infections was shortened 50% after January 28^th^ based on scenario 1.* ***Scenario 3*** *assumed the asymptomatic infections were not controlled (i.e.* $1/{\mathcal{A}_{1}^{i}}=0$*) based on scenario 2. The Y-axis on the left denoted the numbers of daily new infected and the Y-axis on the right represented the proportion of the transmission among daily new infected caused by asymptomatic infections. The 95% credible intervals for simulated numbers are shown for each scenario.*

To visualize the differences in the contribution rate in Figure 5 among different age groups, we proposed seven scenarios of simulations to evaluate the potential outcome if one age group was immune to COVID-19 by the vaccine (Figure S8). If the vaccine could significantly decrease the susceptibility and thereby transmissibility due to not being infected, the numbers of all infections decreased 2.09%, 3.95%, 26.27%, 59.89%, 66.33%, 79.35%, 55.44% in each age group, respectively.


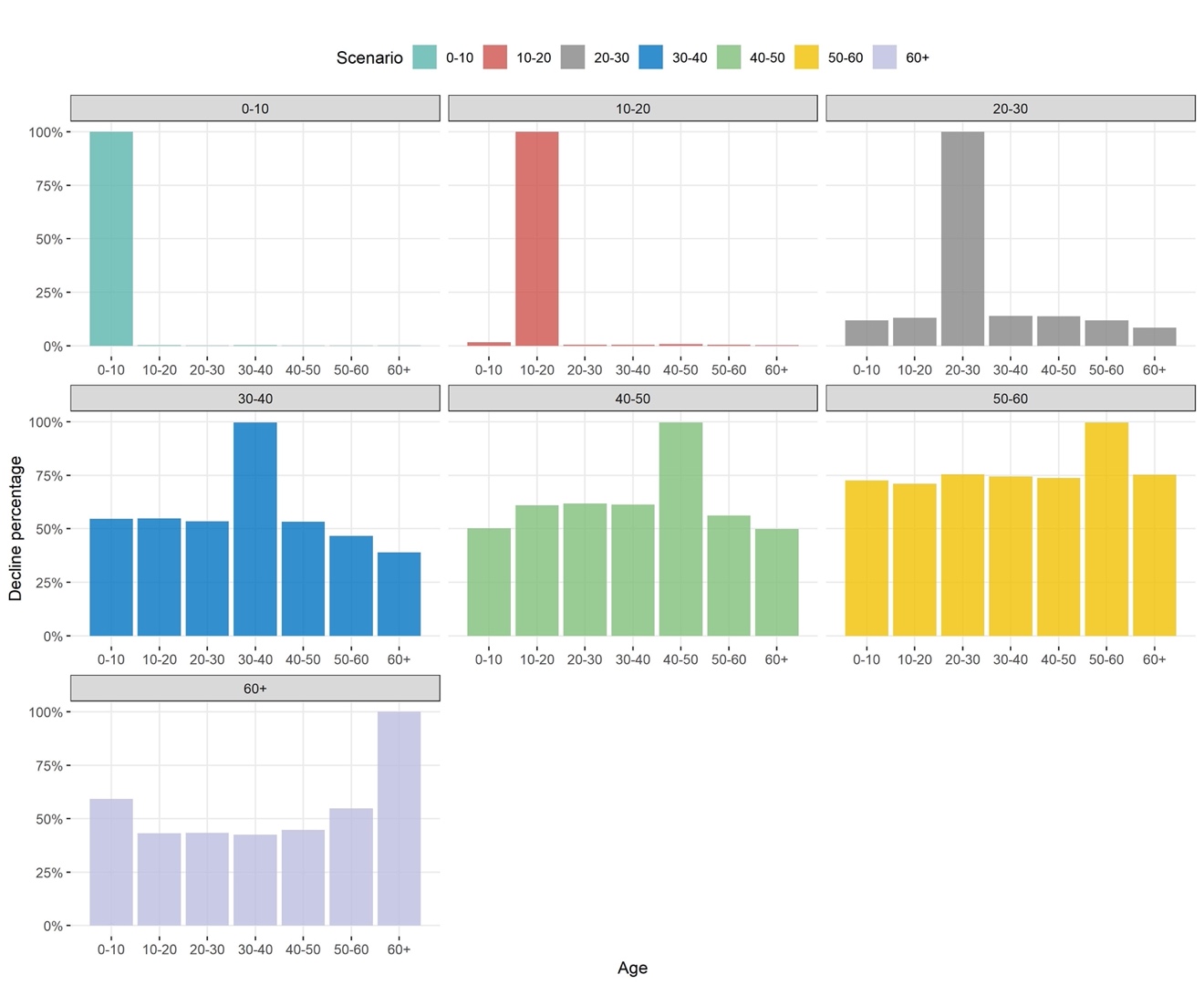


*Figure S8. The simulated decline percentages of all infected individuals in different age groups (the population except the susceptible individuals) until February 22^nd^, 2020 compared with the original estimates in a different scenario. Each scenario assumed one of the age groups was immune to COVID-19, i.e. not susceptible and transmissible to COVID-19, respectively.*

# Sensitivity analyses

## Age-varying transmissibility

The robustness of our model estimates was verified by further sensitivity analyses. By varying the contact matrices, we simulated modified data to validate our estimates of transmissibility.


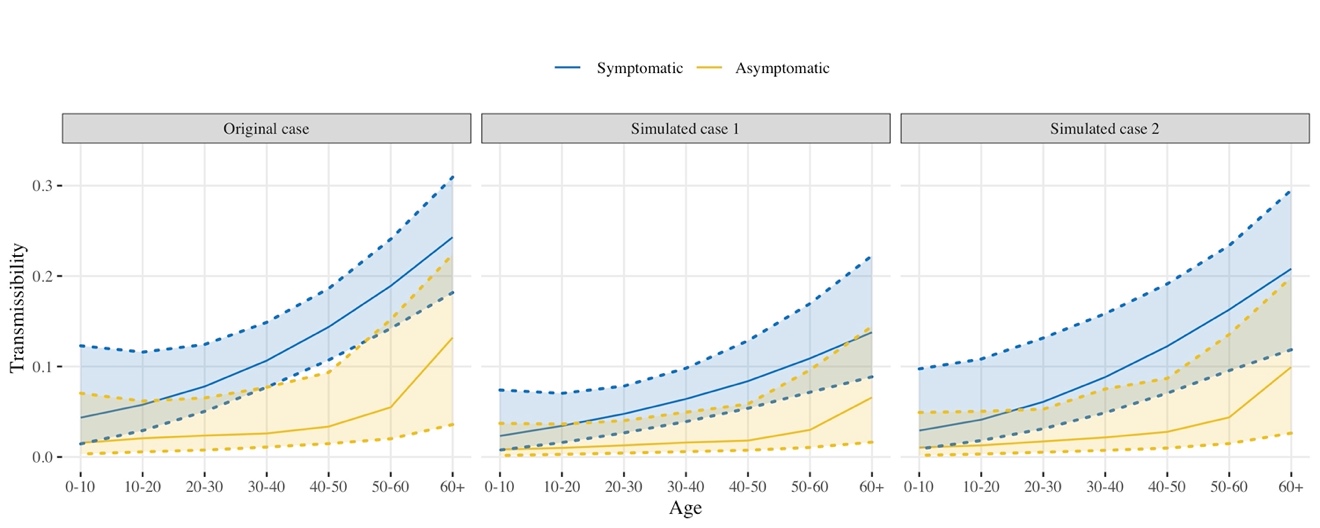


*Figure S9.* *The estimated transmissibility and 95% credible intervals for each age group. Original case: model was fitted with the original contact matrices. Simulated case 1 and 2:* model was fitted with two modified contact matrices, respectively. *Each element of the modified contact matrices was contaminated by a mean-zero Gaussian noise with standard deviance 0.1. By repeating the process of adding noise twice, four modified contact matrices were generated.*

Contact matrices was important to model estimations, and changes of values certainly would influence the estimates. However, in the sensitivity analysis (Figure S9), we found that the trend of transmissibility by age groups did not change.

Besides, noting that the posterior distribution of $\beta_{2}$ in Figure S4 (E) was far from 1, we proposed further sensitivity analyses for the estimated transmissibility to examine the impact of the contact matrices on the outbreak period. We presented the result in Figure S10, using the contact matrices during the outbreak period from Shanghai and Wuhan, respectively. For these sensitivity analyses, we assumed that $\beta_{2}$ was fixed and taken as 1, i.e., we did not introduce the additional correction parameter $\beta_{2}$ and simply explored two fixed versions of contact matrices during the outbreak as sensitivity checks. In Figure S10, we noticed that the trends of the transmissibility by age groups were similar to the originally estimated result for both contact matrices from Shanghai and Wuhan, suggesting that the main pattern of the transmissibility may not be sensitive to different contact matrices during the outbreak and the parameter $\beta_{2}$.

*
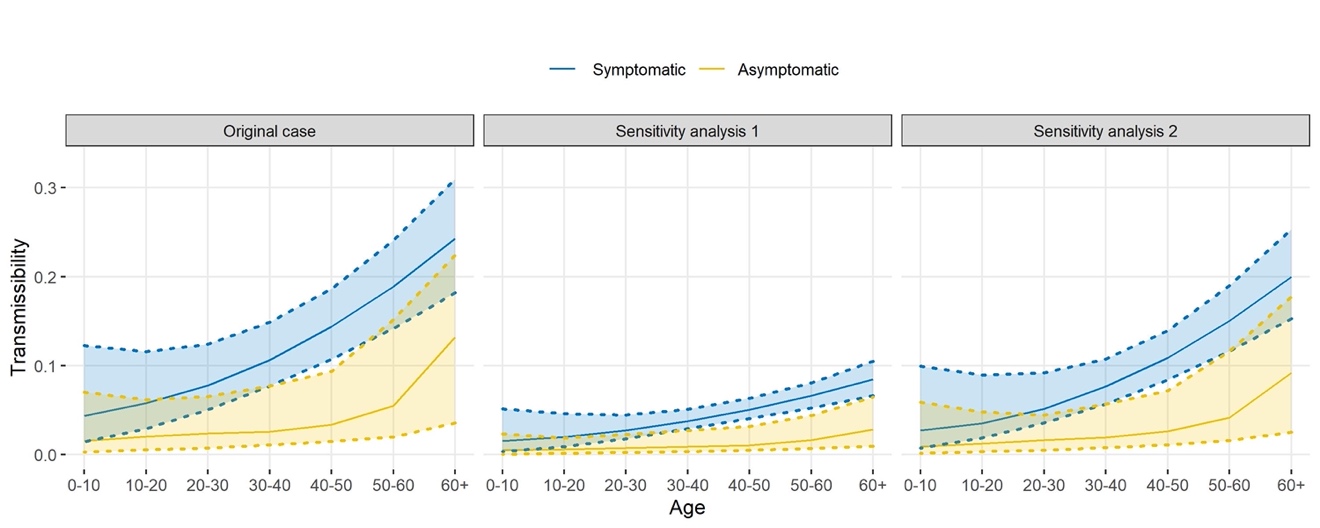
*

*Figure S10. The estimated transmissibility and 95% credible intervals for each age group. Original case: model was fitted with the original contact matrices during the outbreak period. Sensitivity analysis case 1 and 2: model was fitted with two contact matrices on the outbreak period from the Shanghai and Wuhan, respectively, where the correction parameter* $\beta_{2}$ *was assumed to be fixed and taken as 1.*

Next, we further explored different contact matrix structures by altering the contact matrices during both baseline and outbreak periods. The new contact matrices for these additional analyses were generated by resampling without replacement from the original contact matrices in Shanghai. By using the original contact matrix during the baseline period, we found that the estimated transmissibility trends were not sensitive to different structures of the contact matrix during the lockdown (outbreak) period. In Figure S11, all three panels showed similar results of the association between transmissibility and age for both symptomatic and asymptomatic transmissibility.

*
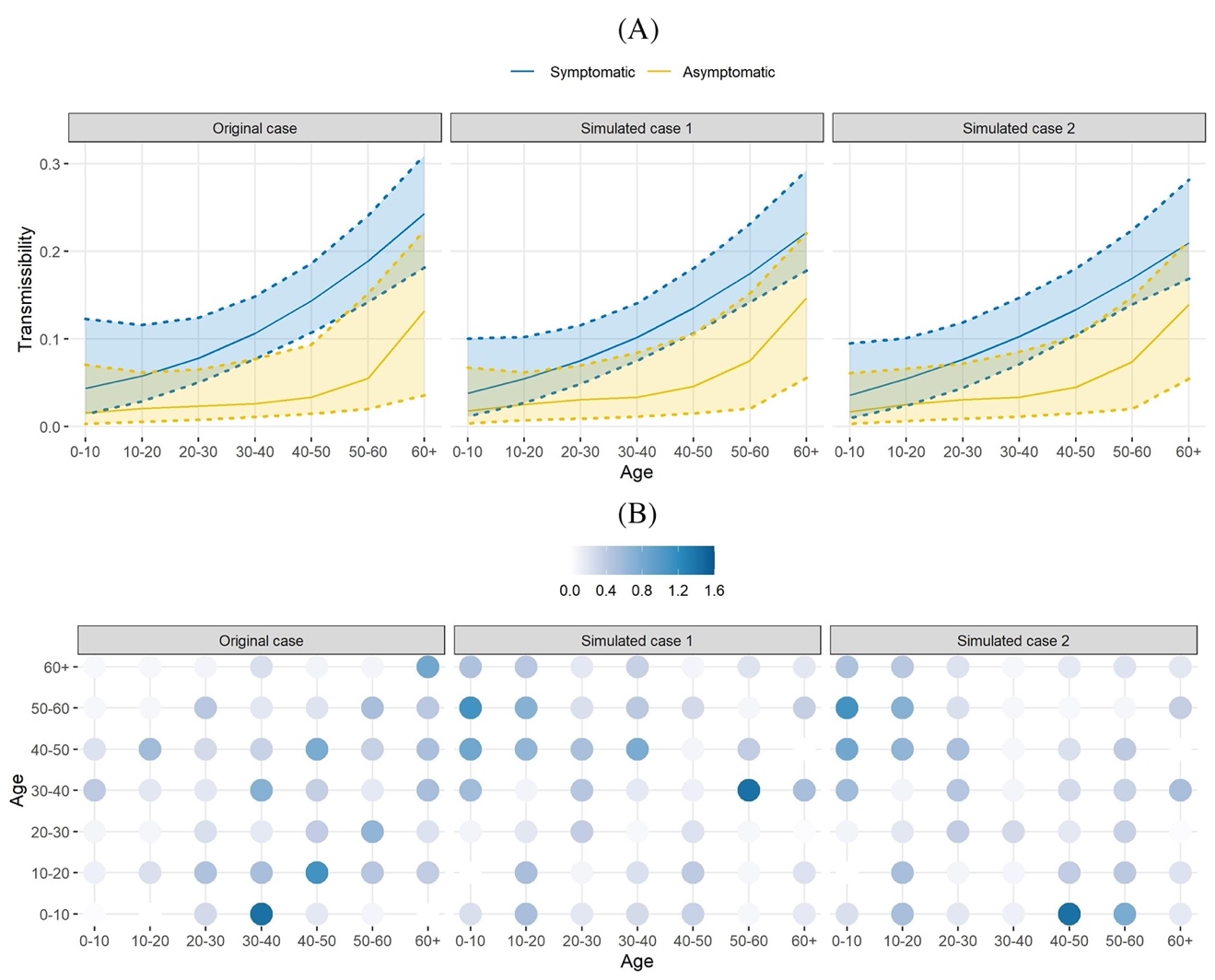
*

*Figure S11. The estimated transmissibility and 95% credible intervals for each age group (A) and the used contact matrices on the outbreak period (B). Original case: model was fitted with the original contact matrices on the outbreak period with the correction parameters* $\beta_{1} and \beta_{2}$ *estimated from data. Simulated case 1 and 2: model was fitted with two contact matrices on the outbreak period by resampling the original contact matrices on the outbreak period from the Shanghai, respectively. For these cases, their corresponding contact matrices were illustrated in panel B (where the shades of color represent different contact numbers). The correction parameters* $\beta_{1} and \beta_{2}$ *were estimated from data.*

We then resampled the original contact matrices in Shanghai during the baseline period to obtain different matrix structures prior to the lockdown/outbreak period. We repeated similar analyses to assess the impact of the baseline contact matrix. In Figure S12, three panels showed inconsistent results of the association between transmissibility and age. Therefore, age-specific transmissibility likely depends on the contact matrix, and in our data example, particularly during the baseline period.

*
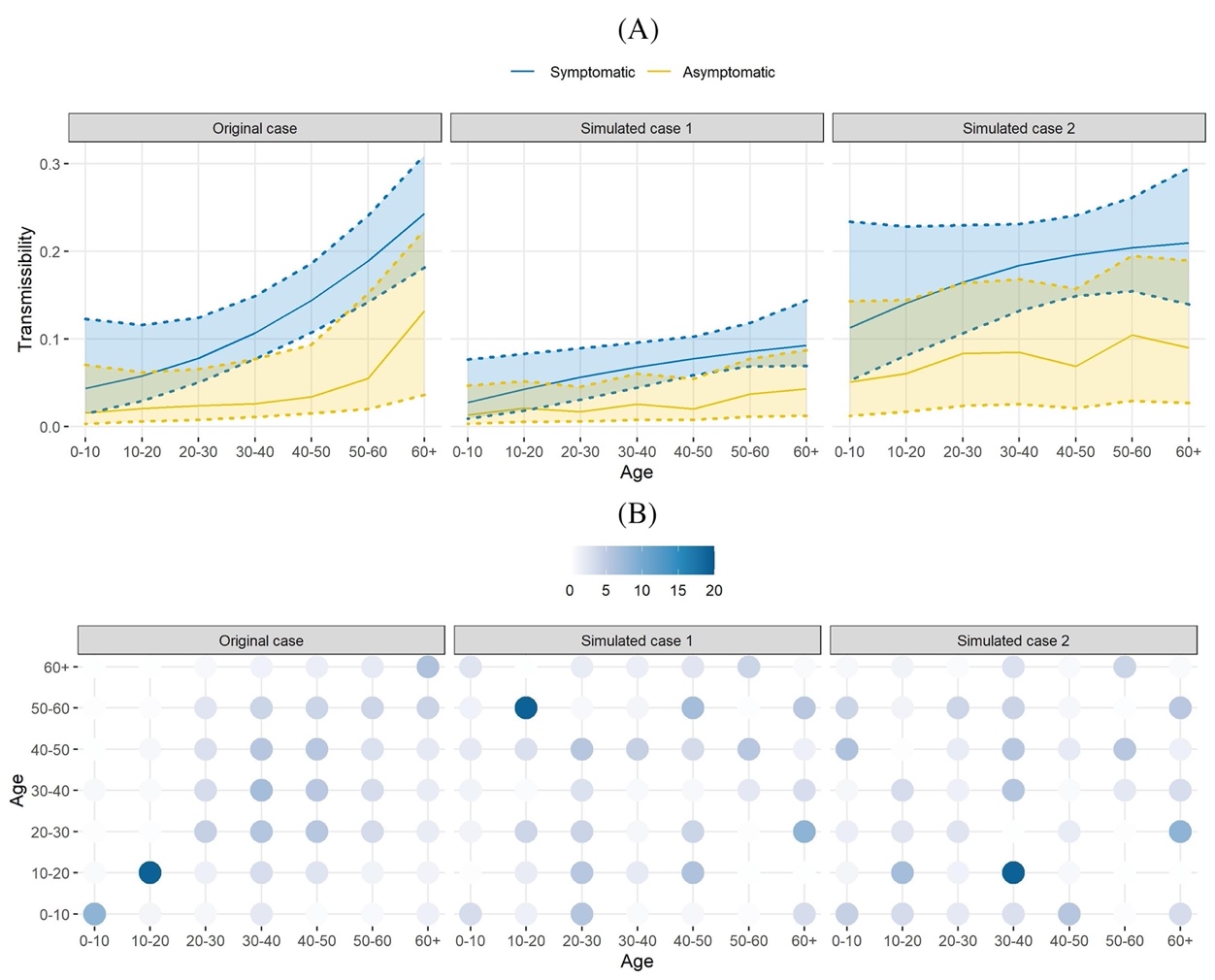
*

*Figure S12. The estimated transmissibility and 95% credible intervals for each age group (A) and the used contact matrices on the baseline period (B). Original case: model was fitted with the original contact matrices on the baseline period. Simulated case 1 and 2: model was fitted with two contact matrices on the baseline period by resampling the original contact matrices on the baseline period from Shanghai, respectively. For these cases, their corresponding contact matrices were illustrated in panel B (where the shades of color represent different contact numbers). The correction parameters* $\beta_{1} and \beta_{2}$ *wee estimated from data.*

These additional results suggested that our estimates were more sensitive to the variations of the contact structure during the baseline period (prior to the lockdown), but less so to those during the lockdown period. We suspected the lockdown strongly suppressed the outbreak in our data example, resulting in limited transmission during this period. Meanwhile, due to the incubation period, most cases that occurred during the lockdown period could be attributed to exposures prior to that period. Consequently, our estimates likely relied more on contact data at the baseline (prior to the lockdown period) and were not as sensitive to the contact matrix during the lockdown period.

## Unconfirmed numbers

We assumed the surveillance had detected most symptomatic cases but had missed some asymptomatic infections. Therefore, for the estimates of the proportion of asymptomatic infections and unconfirmed asymptomatic infections for seven age groups, we added sensitivity analyses by increasing the number of daily confirmed asymptomatic infections of over 30 years old age groups (Figure S13). Results were similar across different settings.


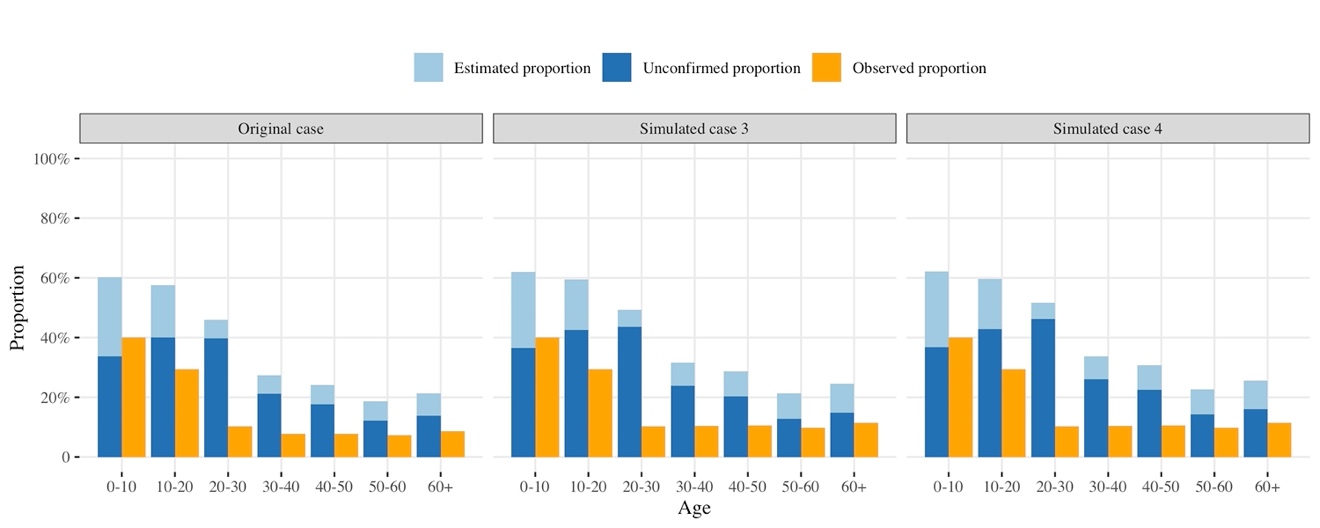


*Figure S13. The estimated proportion of asymptomatic infections and unconfirmed asymptomatic infections for seven age groups by the original data and* *simulated data. Original case: model was fitted with the original data. Simulated case 3 and 4:* model was fitted with two simulated data, respectively. The first simulated data had the numbers of the daily new confirmed asymptomatic infections over 30 years old increased by 20%. The second simulated data had the numbers of the daily new confirmed asymptomatic infections over 30 years old increased by 40%.

## Number of newly infected caused by different transmissions

We also performed sensitivity analyses for the burden of transmission (Figure S14). To account for the potential miss in the detection of asymptomatic infection, we fitted the mode with two simulated data, respectively. The first and second simulated data had 50% and 100% more number of the daily new confirmed asymptomatic infections, respectively. The results of the relative transmission burden from sensitivity analyses were similar to the main result.


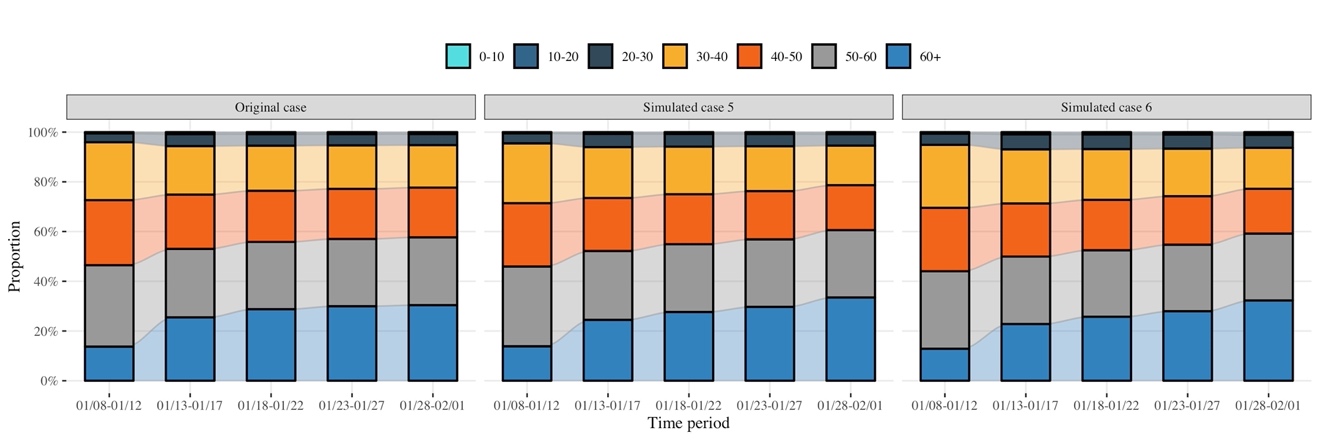


*Figure S14. The estimated burden of transmission caused by different ages by the original data and simulated data from setting C, where simulated cases 5 and 6 represent that the numbers of the daily new confirmed asymptomatic infections from all age groups increased by 50% and 100%, respectively.*

# References

1. **Kim SE, *et al.*** Viral kinetics of SARS-CoV-2 in asymptomatic carriers and presymptomatic patients. *International journal of infectious diseases: IJID: official publication of the International Society for Infectious Diseases* 2020; **95**: 441–443.

2. **Long Q-X, *et al.*** Clinical and immunological assessment of asymptomatic SARS-CoV-2 infections. *Nature Medicine* Nature Publishing Group, 2020; **26**: 1200–1204.

3. **Han MS, *et al.*** Viral RNA Load in Mildly Symptomatic and Asymptomatic Children with COVID-19, Seoul, South Korea. *Emerging Infectious Diseases* 2020; **26**: 2497–2499.

4. **Lee S, *et al.*** Clinical Course and Molecular Viral Shedding Among Asymptomatic and Symptomatic Patients With SARS-CoV-2 Infection in a Community Treatment Center in the Republic of Korea. *JAMA Internal Medicine* 2020; **180**: 1447–1452.

5. **Davies NG, *et al.*** Age-dependent effects in the transmission and control of COVID-19 epidemics. *Nature Medicine* Nature Publishing Group, 2020; **26**: 1205–1211.

6. **Tian T, *et al.*** Evaluate the Risk of Resumption of Business for the States of New York, New Jersey and Connecticut via a Pre-Symptomatic and Asymptomatic Transmission Model of COVID-19. *Journal of Data Science* School of Statistics, Renmin University of China, 2021; Published online: 20 February 2021.

7. **Zhang J, *et al.*** Changes in contact patterns shape the dynamics of the COVID-19 outbreak in China. *Science (New York, N.Y.)* 2020; **368**: 1481–1486.

8. **Zhang J, *et al.*** Patterns of human social contact and contact with animals in Shanghai, China. *Scientific Reports* Nature Publishing Group, 2019; **9**: 15141.

9. **Gao M, *et al.*** A study on infectivity of asymptomatic SARS-CoV-2 carriers. *Respiratory Medicine* 2020; **169**: 106026.

10. **Luo L, *et al.*** Contact Settings and Risk for Transmission in 3410 Close Contacts of Patients With COVID-19 in Guangzhou, China : A Prospective Cohort Study. *Annals of Internal Medicine* American College of Physicians, 2020; **173**: 879–887.

11. **Zhou F, *et al.*** Clinical course and risk factors for mortality of adult inpatients with COVID-19 in Wuhan, China: a retrospective cohort study. *The Lancet* 2020; **395**: 1054–1062.

12. **Wikle CK, Berliner LM**. A Bayesian tutorial for data assimilation. *Physica D: Nonlinear Phenomena* 2007; **230**: 1–16.

13. **Bureau of Statistics**. *Zhejiang Provincial Bureau of Statistics: Sixth Census Data*. 2014(http://tjj.zj.gov.cn/art/2014/9/3/art_1530851_20980968.html). Accessed 30 July 2020.

14. **Lauer SA, *et al.*** The Incubation Period of Coronavirus Disease 2019 (COVID-19) From Publicly Reported Confirmed Cases: Estimation and Application. *Annals of Internal Medicine* American College of Physicians, 2020; **172**: 577–582.

15. **Backer JA, Klinkenberg D, Wallinga J**. Incubation period of 2019 novel coronavirus (2019-nCoV) infections among travellers from Wuhan, China, 20-28 January 2020. *Euro Surveillance: Bulletin Europeen Sur Les Maladies Transmissibles = European Communicable Disease Bulletin* European Centre for Disease Prevention and Control, 2020; **25**Published online: 2020.doi:10.2807/1560-7917.ES.2020.25.5.2000062.

16. **Kong D, *et al.*** Pre-symptomatic transmission of novel coronavirus in community settings. *Influenza and Other Respiratory Viruses* 2020; **14**: 610–614.

17. **Wei WE, *et al.*** Presymptomatic Transmission of SARS-CoV-2 — Singapore, January 23–March 16, 2020. *Morbidity and Mortality Weekly Report* 2020; **69**: 411–415.

18. **Elfessi A, Reineke DM**. A Bayesian Look at Classical Estimation: The Exponential Distribution. *Journal of Statistics Education* Taylor & Francis, 2001; **9**Published online: 1 January 2001.doi:10.1080/10691898.2001.11910648.

19. **Givens GH, Hoeting JA**. *Computational Statistics*. John Wiley & Sons, 2012.

20. **Andrieu C, *et al.*** An Introduction to MCMC for Machine Learning. *Machine Learning* 2003; **50**: 5–43.

21. **Knaus J, *et al.*** Easier parallel computing in R with snowfall and sfCluster. *The R Journal* 2009; **1**: 54–59.

22. **Gelman A, Meng X-L, Stern H**. POSTERIOR PREDICTIVE ASSESSMENT OF MODEL FITNESS VIA REALIZED DISCREPANCIES. *Statistica Sinica* Institute of Statistical Science, Academia Sinica, 1996; **6**: 733–760.

1. The mean of the susceptibility of “60+” is calculated as the average of the mean for age groups “60-69” and “70+” in that study. [↑](#footnote-ref-1)
