# Supplementary figures and images for: Transmission roles of symptomatic and asymptomatic COVID-19 cases: a modelling study
Source: Epidemiol Infect. 2022 Sep 27;150:e171. doi: 10.1017/S0950268822001467 (PMC9588416; doi:10.1017/S0950268822001467)

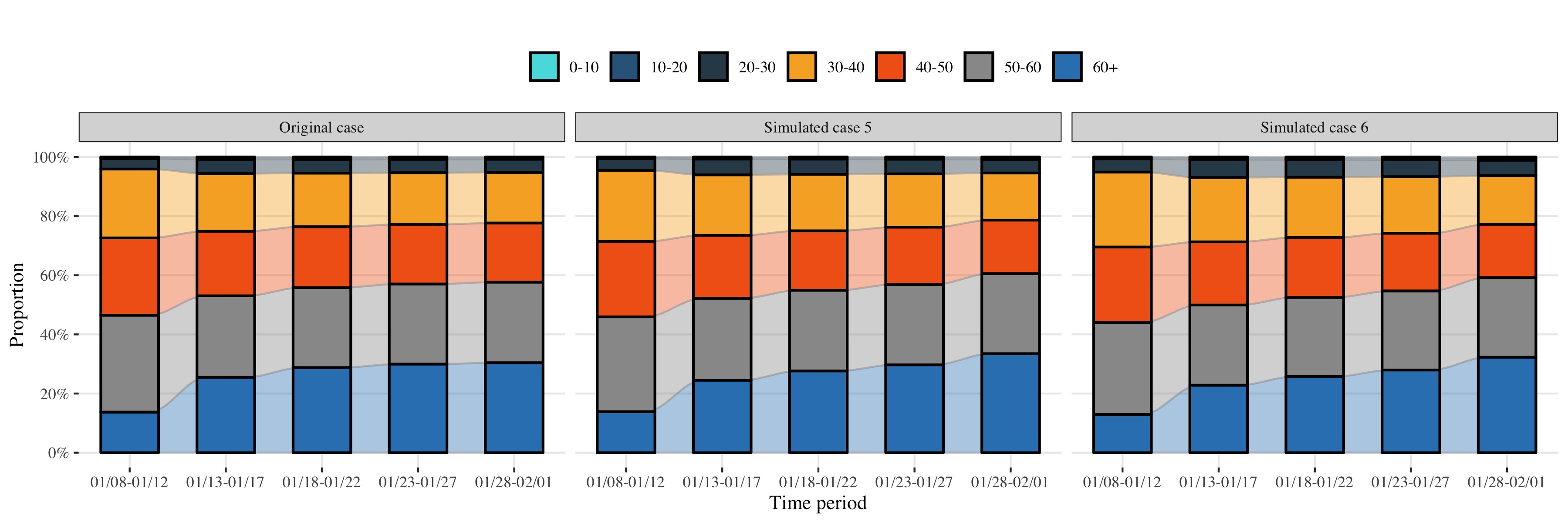

Supplement: Supplementary file 1 [file S0950268822001467sup001.zip › S0950268822001467sup002.jpg]

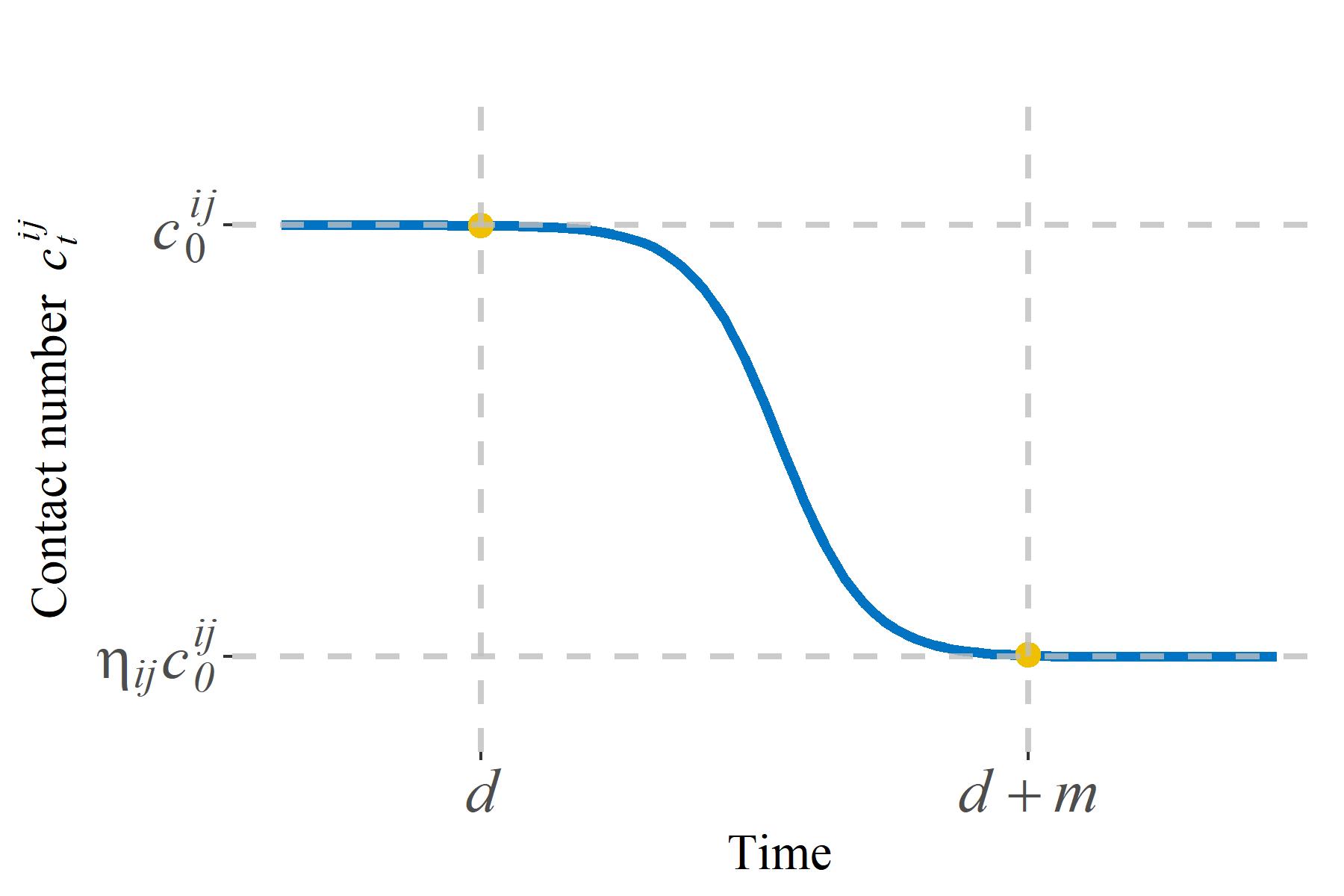

Supplement: Supplementary file 1 [file S0950268822001467sup001.zip › S0950268822001467sup003.jpg]

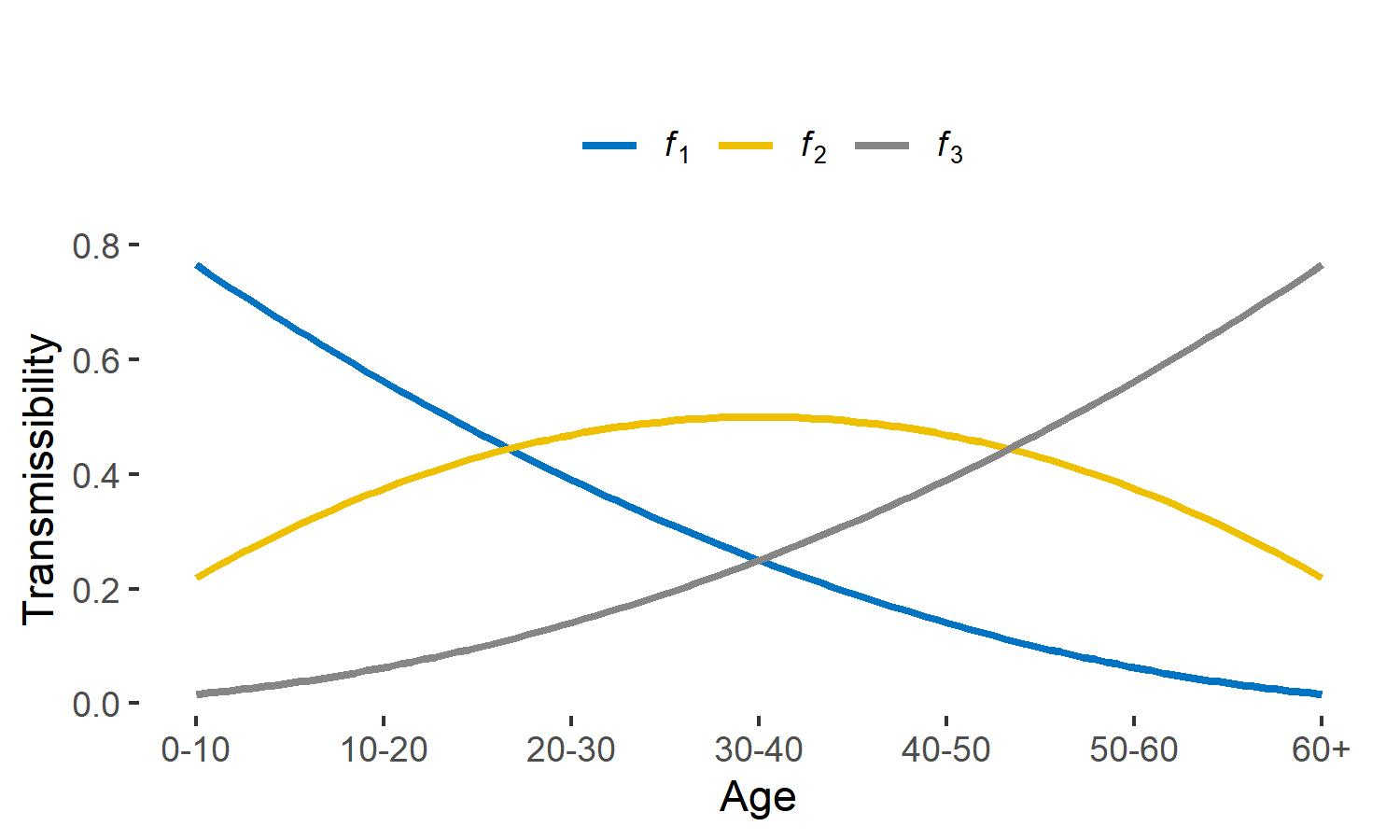

Supplement: Supplementary file 1 [file S0950268822001467sup001.zip › S0950268822001467sup004.jpg]

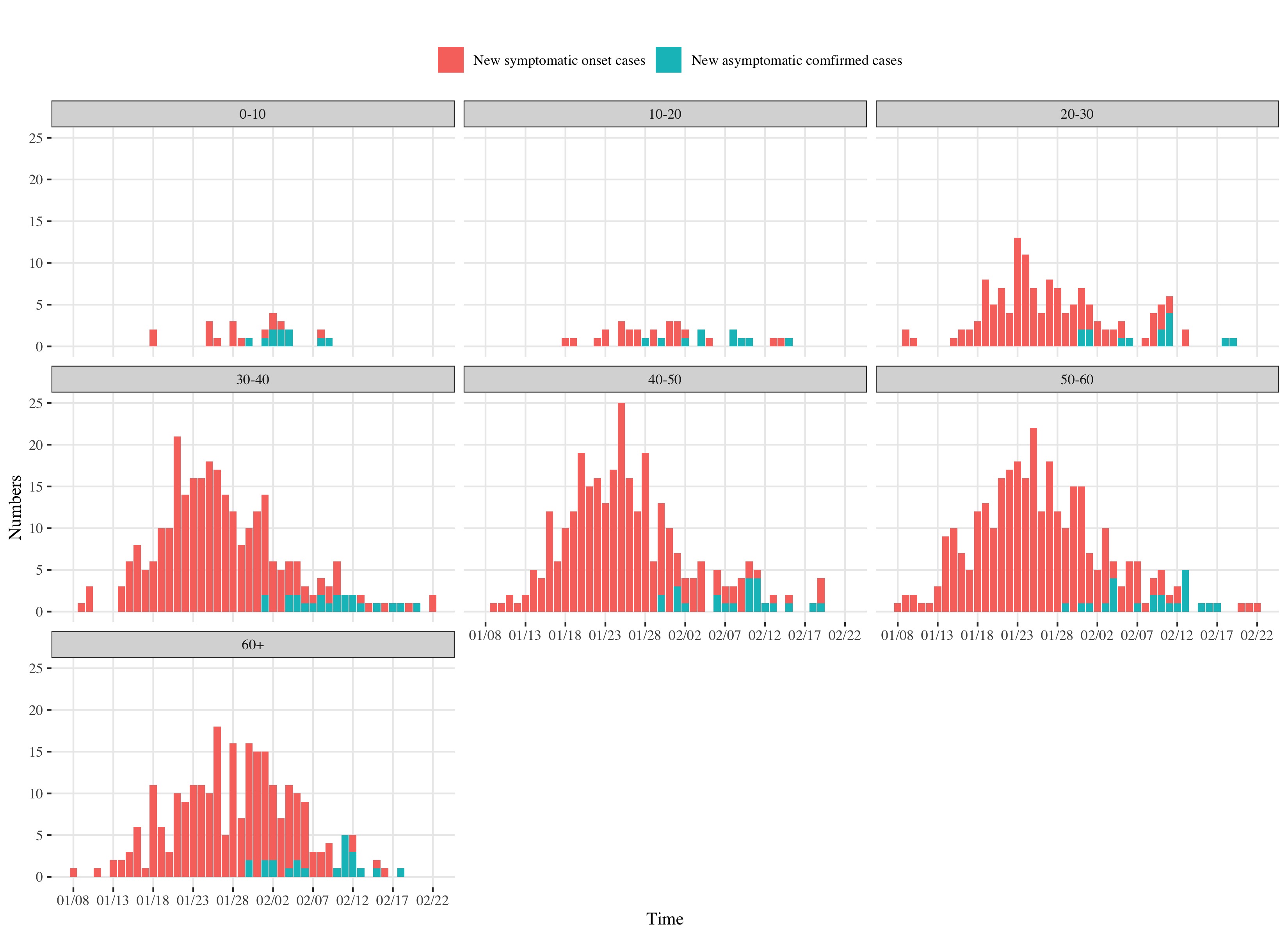

Supplement: Supplementary file 1 [file S0950268822001467sup001.zip › S0950268822001467sup005.jpg]

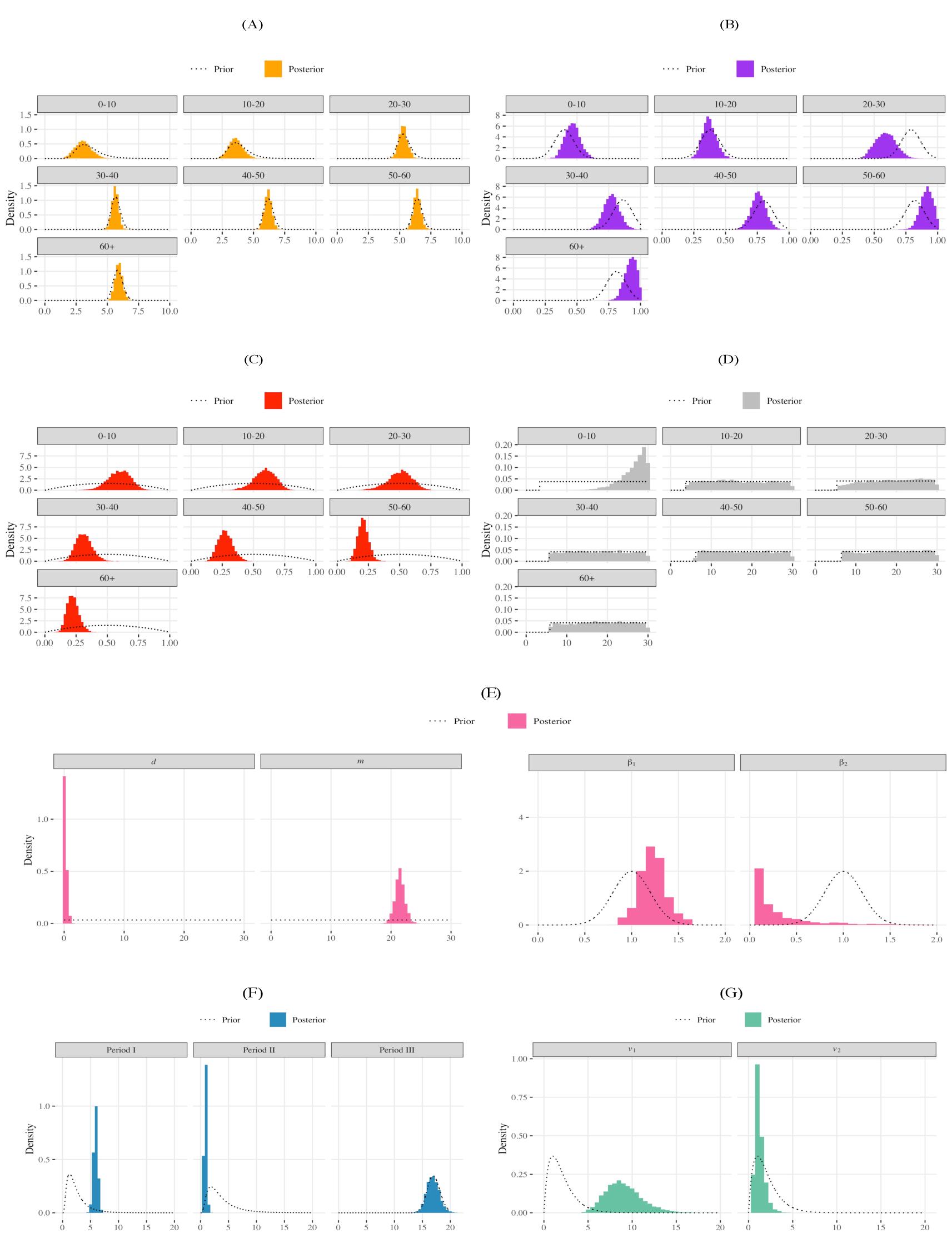

Supplement: Supplementary file 1 [file S0950268822001467sup001.zip › S0950268822001467sup006.jpg]

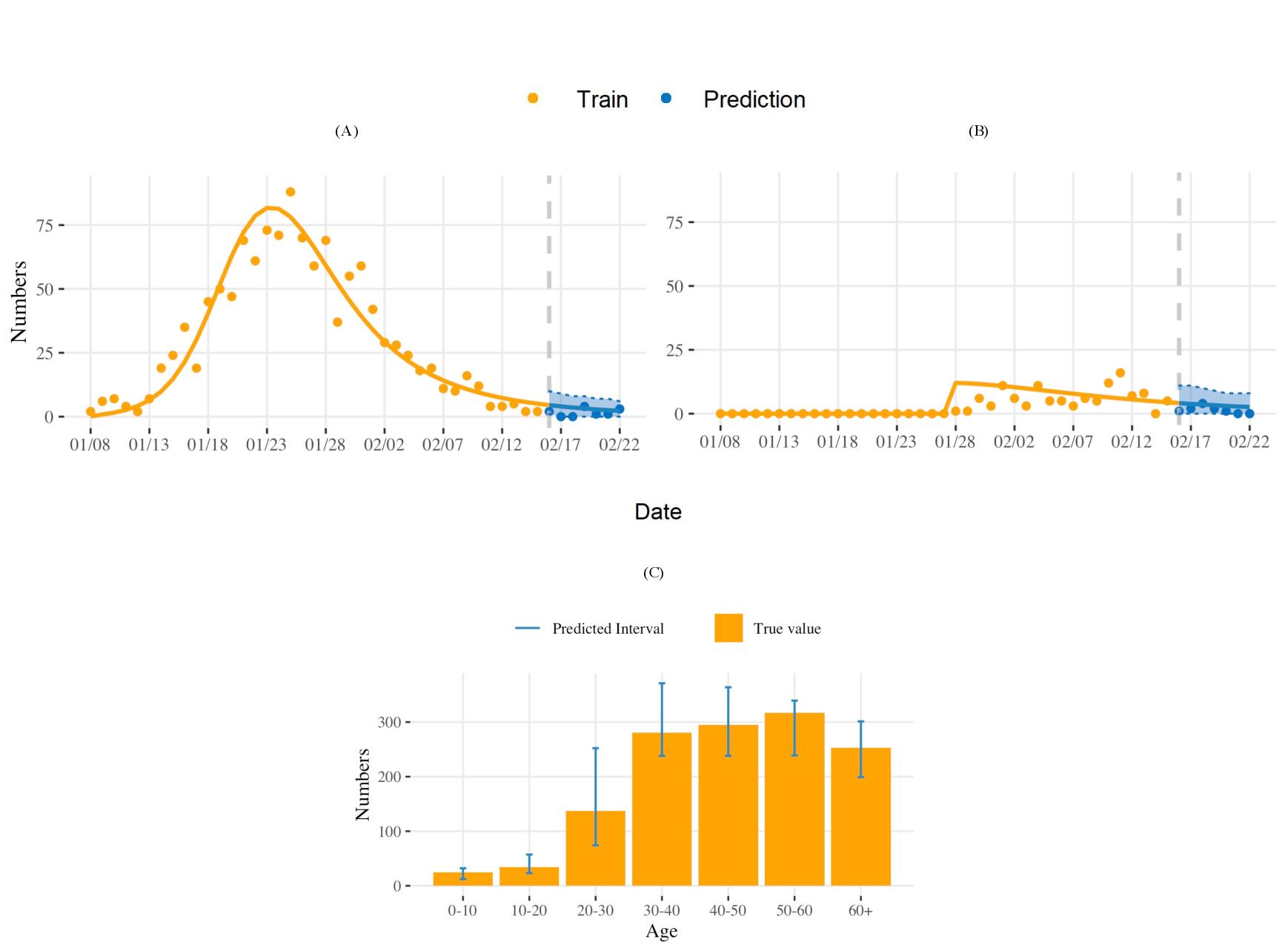

Supplement: Supplementary file 1 [file S0950268822001467sup001.zip › S0950268822001467sup007.jpg]

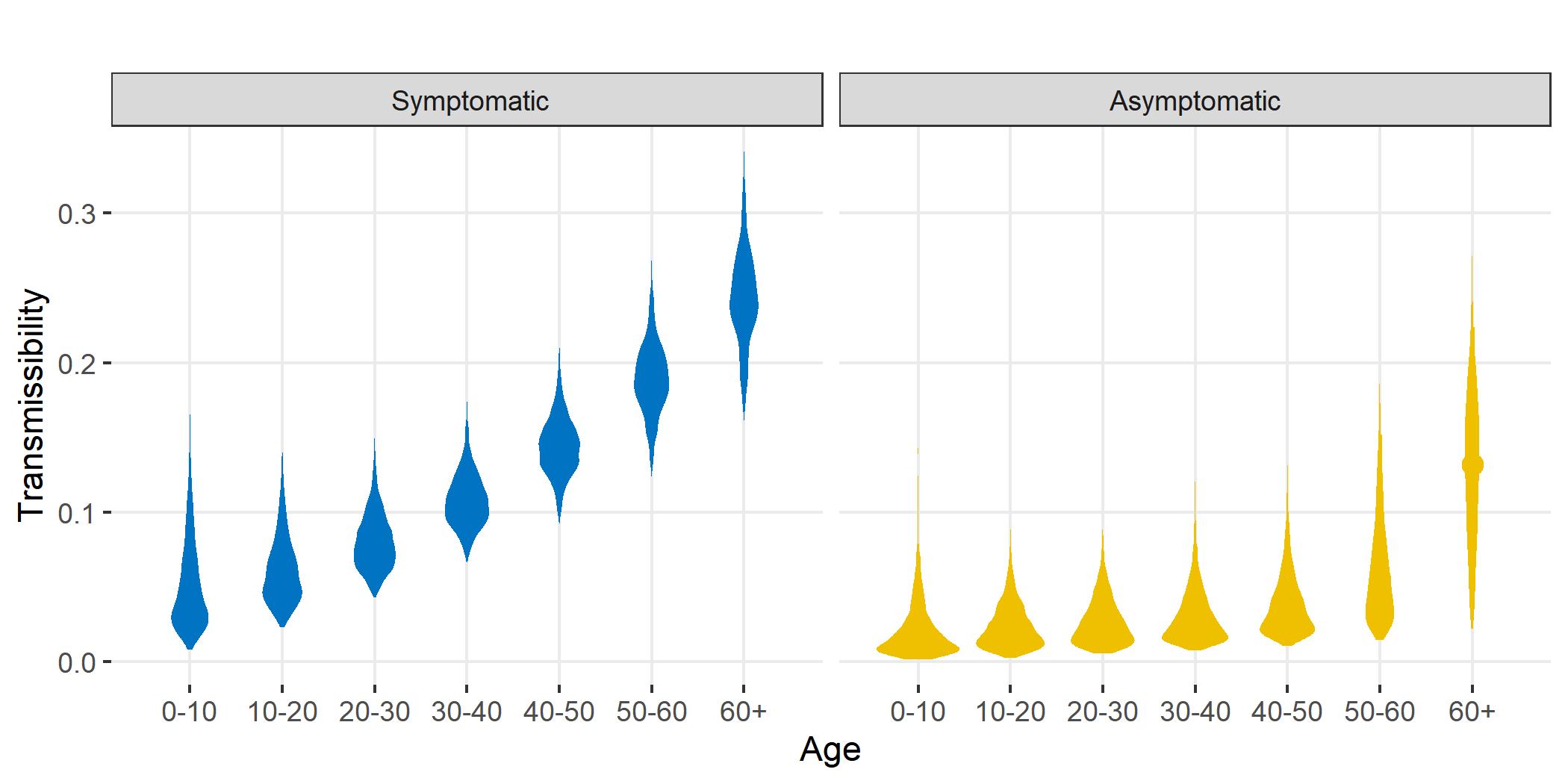

Supplement: Supplementary file 1 [file S0950268822001467sup001.zip › S0950268822001467sup008.jpg]

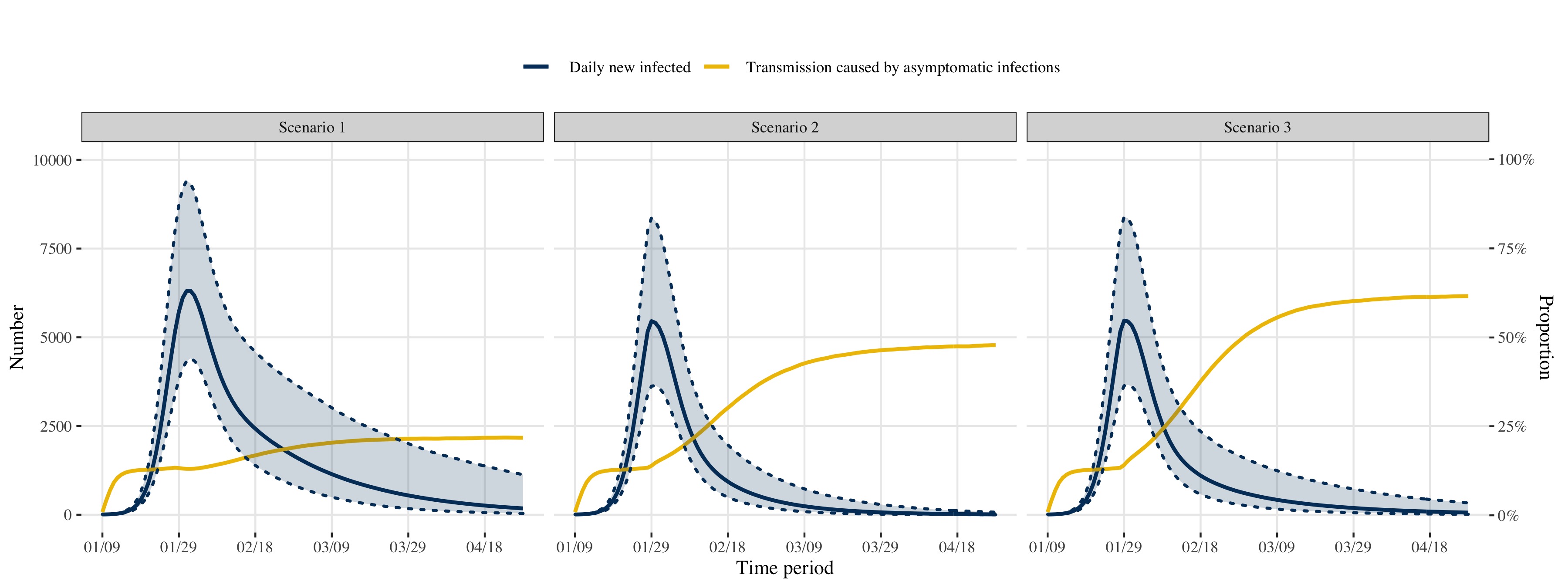

Supplement: Supplementary file 1 [file S0950268822001467sup001.zip › S0950268822001467sup009.jpg]

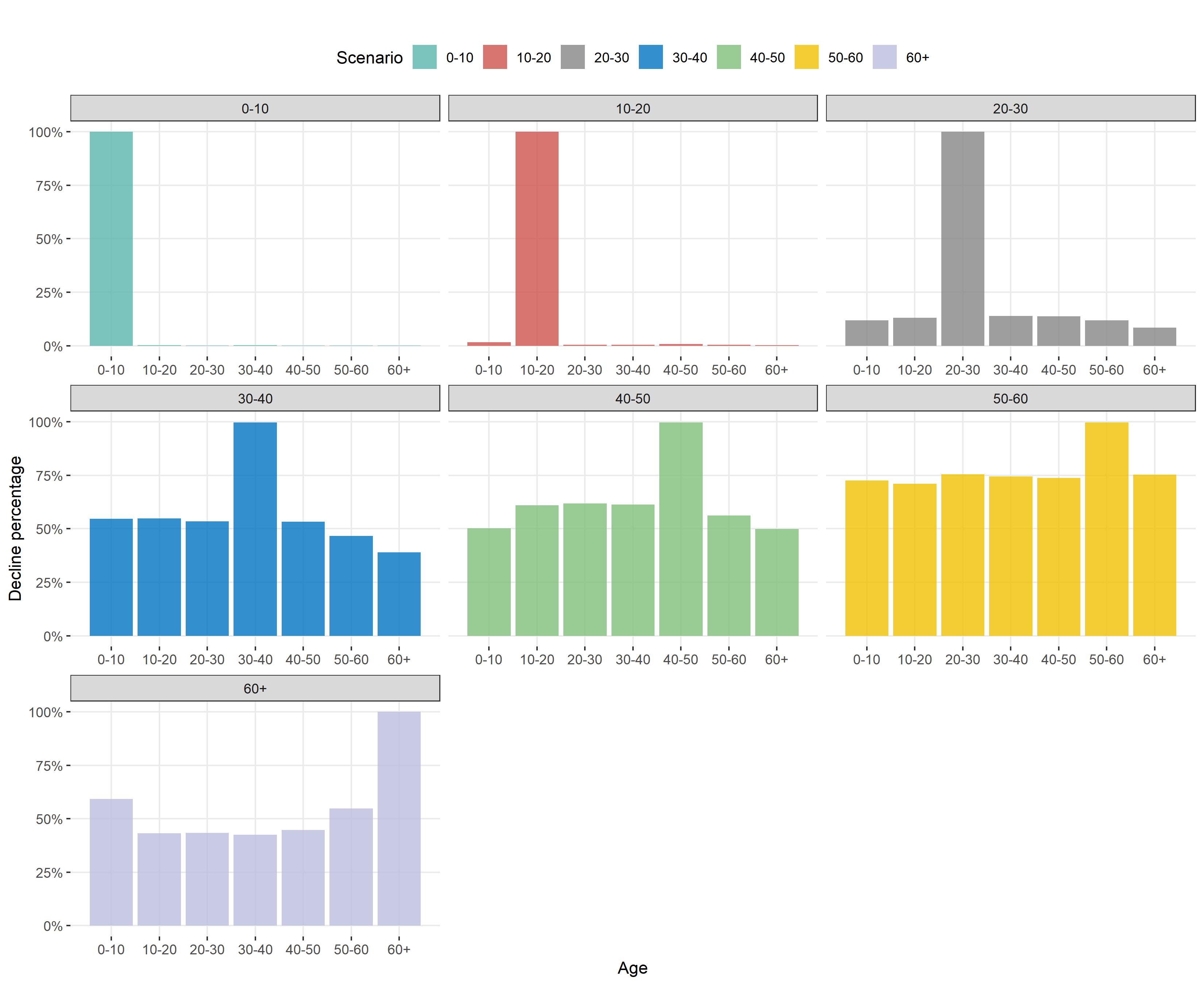

Supplement: Supplementary file 1 [file S0950268822001467sup001.zip › S0950268822001467sup010.jpg]

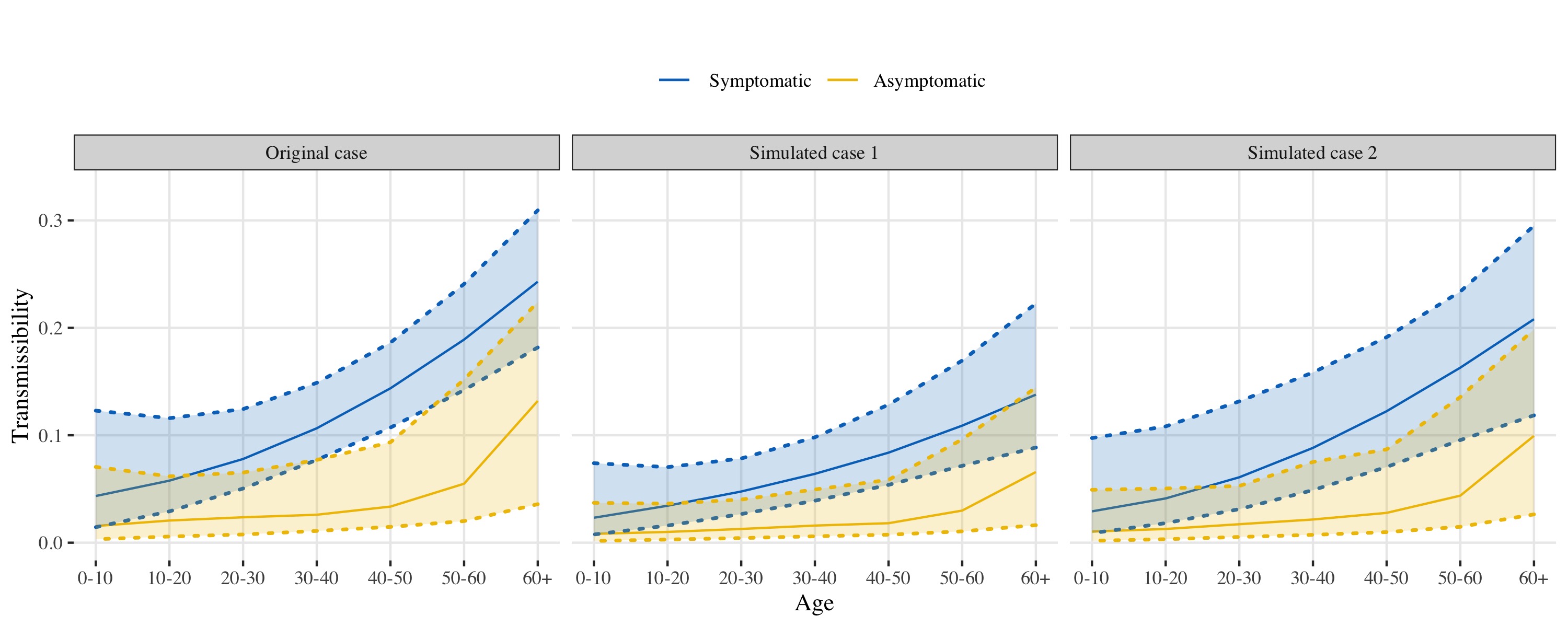

Supplement: Supplementary file 1 [file S0950268822001467sup001.zip › S0950268822001467sup011.jpg]

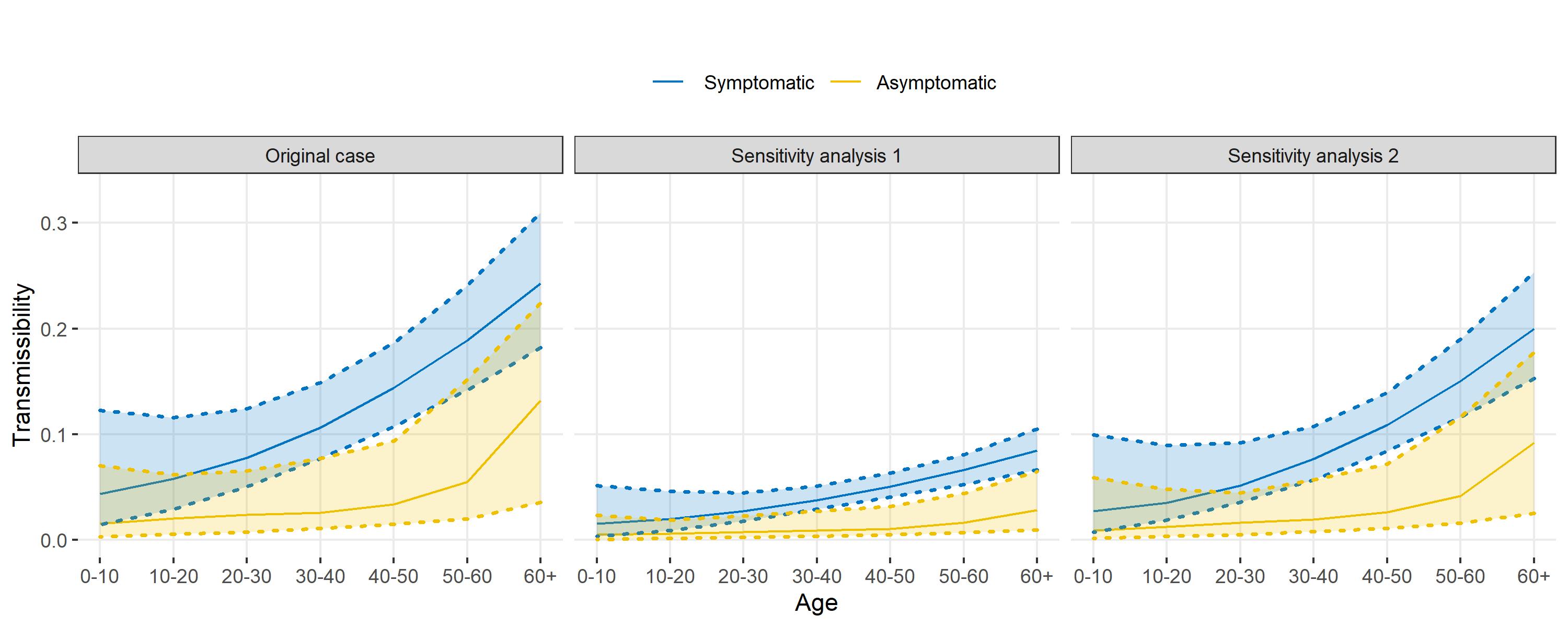

Supplement: Supplementary file 1 [file S0950268822001467sup001.zip › S0950268822001467sup012.jpg]

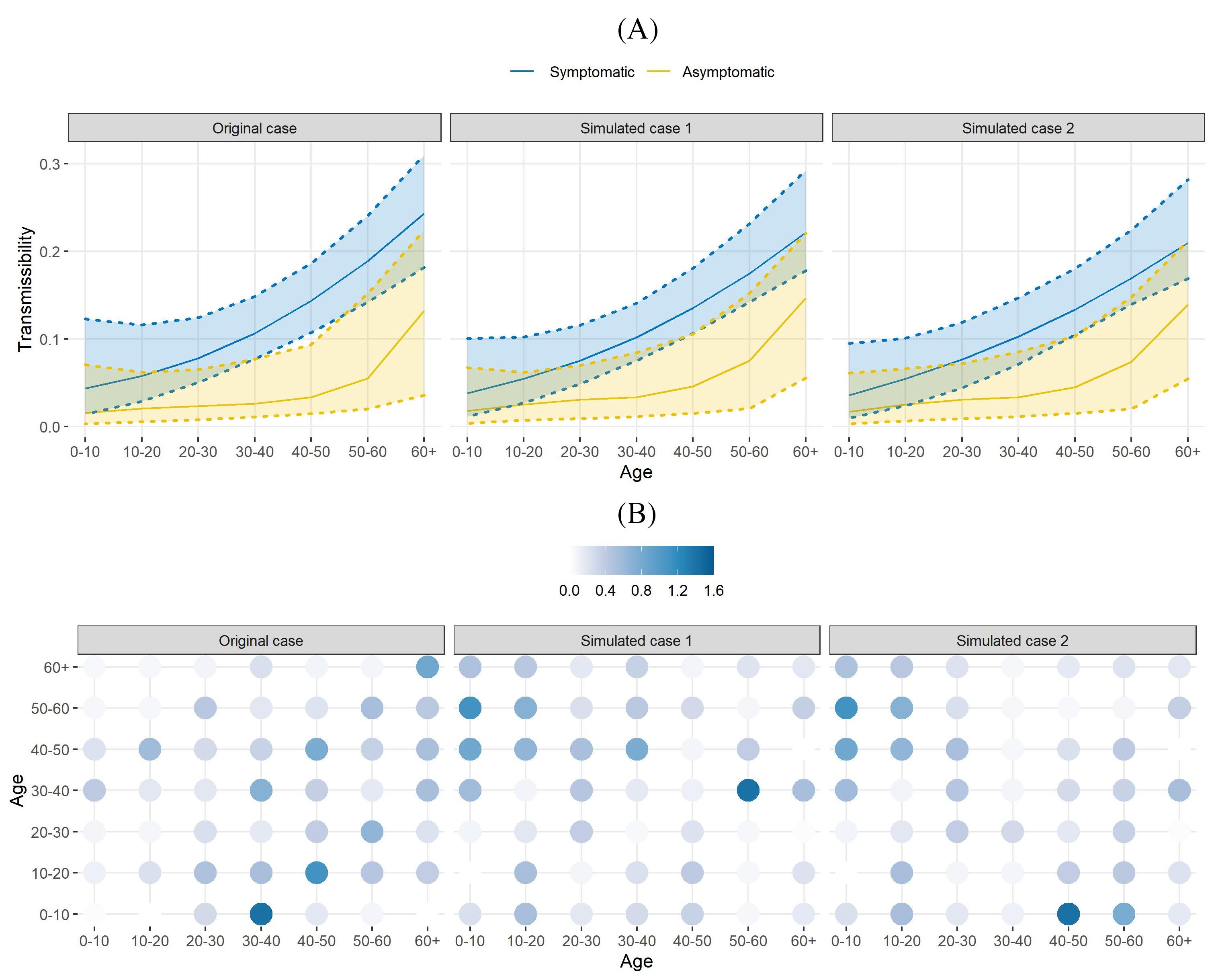

Supplement: Supplementary file 1 [file S0950268822001467sup001.zip › S0950268822001467sup013.jpg]

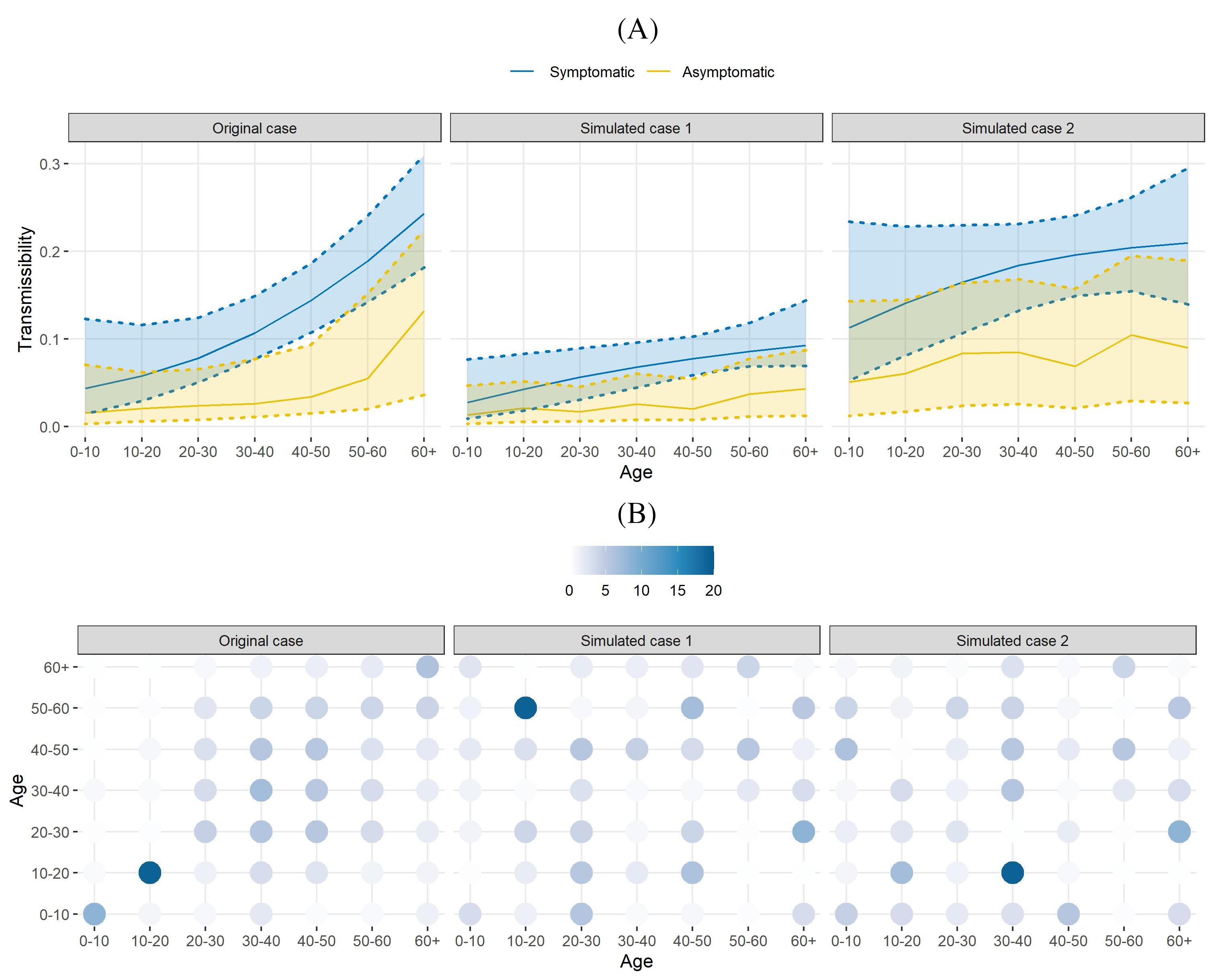

Supplement: Supplementary file 1 [file S0950268822001467sup001.zip › S0950268822001467sup014.jpg]

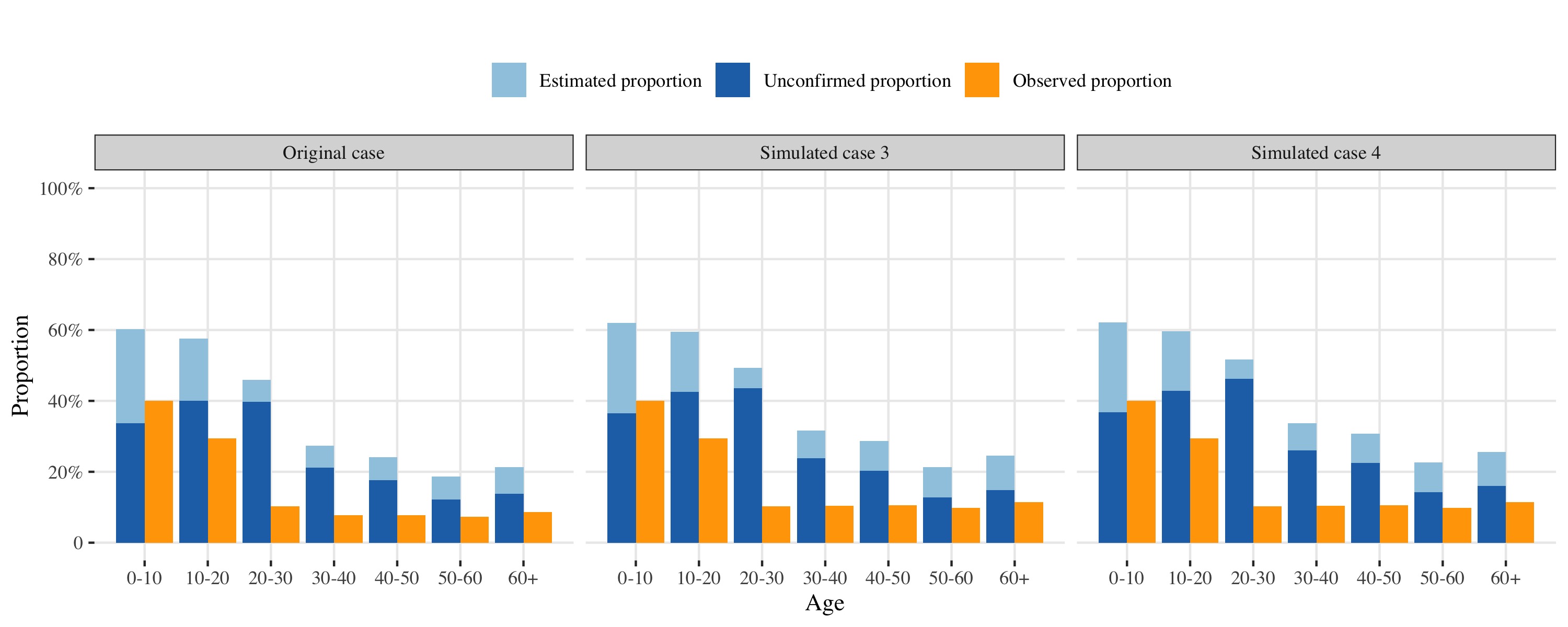

Supplement: Supplementary file 1 [file S0950268822001467sup001.zip › S0950268822001467sup015.jpg]
